# Host genetic variation and specialized metabolites from wheat leaves enriches for phyllosphere *Pseudomonas* spp. with enriched antibiotic resistomes

**DOI:** 10.1093/ismejo/wrae144

**Published:** 2024-07-29

**Authors:** Qian Xiang, Da Lin, Zai-Jun Yang, Rui-Xia Han, Tian-Lun Zhang, Qing-Lin Chen, Dong Zhu, Josep Penuelas, Yong-Guan Zhu

**Affiliations:** Key Laboratory of Urban Environment and Health, Ningbo Urban Environment Observation and Research Station, Institute of Urban Environment, Chinese Academy of Sciences, Xiamen 361021, China; Zhejiang Key Laboratory of Urban Environmental Processes and Pollution Control, CAS Haixi Industrial Technology Innovation Center in Beilun, Ningbo 315830, China; Key Laboratory of Urban Environment and Health, Ningbo Urban Environment Observation and Research Station, Institute of Urban Environment, Chinese Academy of Sciences, Xiamen 361021, China; Zhejiang Key Laboratory of Urban Environmental Processes and Pollution Control, CAS Haixi Industrial Technology Innovation Center in Beilun, Ningbo 315830, China; University of Chinese Academy of Sciences, Beijing 100049, China; Key Laboratory of Southwest China Wildlife Resources Conservation (ministry of education), College of Life Science, China West Normal University, Nanchong, Sichuan 637009, China; Key Laboratory of Urban Environment and Health, Ningbo Urban Environment Observation and Research Station, Institute of Urban Environment, Chinese Academy of Sciences, Xiamen 361021, China; Zhejiang Key Laboratory of Urban Environmental Processes and Pollution Control, CAS Haixi Industrial Technology Innovation Center in Beilun, Ningbo 315830, China; Key Laboratory of Urban Environment and Health, Ningbo Urban Environment Observation and Research Station, Institute of Urban Environment, Chinese Academy of Sciences, Xiamen 361021, China; Zhejiang Key Laboratory of Urban Environmental Processes and Pollution Control, CAS Haixi Industrial Technology Innovation Center in Beilun, Ningbo 315830, China; University of Chinese Academy of Sciences, Beijing 100049, China; Key Laboratory of Urban Environment and Health, Ningbo Urban Environment Observation and Research Station, Institute of Urban Environment, Chinese Academy of Sciences, Xiamen 361021, China; Zhejiang Key Laboratory of Urban Environmental Processes and Pollution Control, CAS Haixi Industrial Technology Innovation Center in Beilun, Ningbo 315830, China; Key Laboratory of Urban Environment and Health, Ningbo Urban Environment Observation and Research Station, Institute of Urban Environment, Chinese Academy of Sciences, Xiamen 361021, China; Zhejiang Key Laboratory of Urban Environmental Processes and Pollution Control, CAS Haixi Industrial Technology Innovation Center in Beilun, Ningbo 315830, China; CSIC, Global Ecology Unit CREAF-CSIC-UAB, Bellaterra, Barcelona 08193, Catalonia, Spain; CREAF, Campus Universitat Autònoma de Barcelona, Cerdanyola del Vallès, Barcelona 08193, Catalonia, Spain; Key Laboratory of Urban Environment and Health, Ningbo Urban Environment Observation and Research Station, Institute of Urban Environment, Chinese Academy of Sciences, Xiamen 361021, China; State Key Laboratory of Urban and Regional Ecology, Research Center for Eco-Environmental Sciences, Chinese Academy of Sciences, Beijing 100085, China

**Keywords:** antibiotic resistance genes, plant-microbe co-evolution, Pseudomonas spp, metabolites, plant genetic variation

## Abstract

Antibiotic resistance in plant-associated microbiomes poses significant risks for agricultural ecosystems and human health. Although accumulating evidence suggests a role for plant genotypes in shaping their microbiome, almost nothing is known about how the changes of plant genetic information affect the co-evolved plant microbiome carrying antibiotic resistance genes (ARGs). Here, we selected 16 wheat cultivars and experimentally explored the impact of host genetic variation on phyllosphere microbiome, ARGs, and metabolites. Our results demonstrated that host genetic variation significantly influenced the phyllosphere resistomes. Wheat genotypes exhibiting high phyllosphere ARGs were linked to elevated *Pseudomonas* populations, along with increased abundances of *Pseudomonas aeruginosa* biofilm formation genes. Further analysis of 350 *Pseudomonas* spp. genomes from diverse habitats at a global scale revealed that nearly all strains possess multiple ARGs, virulence factor genes (VFGs), and mobile genetic elements (MGEs) on their genomes, albeit with lower nucleotide diversity compared to other species. These findings suggested that the proliferation of *Pseudomonas* spp. in the phyllosphere significantly contributed to antibiotic resistance. We further observed direct links between the upregulated leaf metabolite DIMBOA-Glc, *Pseudomonas* spp., and enrichment of phyllosphere ARGs, which were corroborated by microcosm experiments demonstrating that DIMBOA-Glc significantly enhanced the relative abundance of *Pseudomonas* spp. Overall, alterations in leaf metabolites resulting from genetic variation throughout plant evolution may drive the development of highly specialized microbial communities capable of enriching phyllosphere ARGs. This study enhances our understanding of how plants actively shape microbial communities and clarifies the impact of host genetic variation on the plant resistomes.

## Introduction

Microbial communities are considered the primary determinant of the antibiotic resistomes [1, 2]. These communities have coevolved with their plant hosts for over 400 million years, influenced by intrinsic factors such as plant genotype [3, 4], age [5], and species [6], as well as by various biotic and abiotic environmental factors including geographical location [4, 7], soil type [7], climate [8], and insect herbivory [9]. Of particular interest among these factors is the impact of plant genetic variation on microbial communities. For example, genome-wide association studies have shown that different *Arabidopsis* accessions exhibited different microbial communities, and the plant loci responsible for defense and cell wall integrity affect the variation in leaf microbial communities [10, 11]. Over evolutionary and domestication timescales, plant genetic information undergoes functional rearrangements, acquisitions, and losses, driven by natural and artificial selection aimed at enhancing plant fitness and increasing crop yield. Such natural and artificial selection can act on plant traits including tissue structure (e.g. cutin and cuticular wax properties, trichome branching), physiology (e.g. exudates and volatiles), plant defense, and hormone signaling pathways (e.g. auxin), which significantly shape the plant microbial communities and initiate microbe–microbe interactions [5, 12, 13]. For instance, plant-derived benzoxazinoids can function as antibiotics and exert selective pressure on bacterial communities [14]. Understanding how changes in plant genetic information influence the co-evolved plant microbiome is crucial because these changes can alter microbial community composition and function, potentially affecting the dissemination of antibiotic-resistance genes. Given the rising concern over antibiotic resistance, it is essential to explore these genetic interactions to develop strategies for managing and mitigating the spread of resistance within agricultural ecosystems.

Plant metabolites are pivotal in governing interactions between plants and microorganisms, contributing to the active reconstruction of microbial communities [15]. Variations in microbiota among plant species or genotypes are often associated with differences in plant exudates [12, 16, 17]. For instance, altered exudation of defensive compounds (e.g. benzoxazinoids) leads to a reorganization of the root microbiome in mutants compared to wild-type maize [17, 18]. Compared to the rhizosphere, the phyllosphere represents an open system and hosts distinct microbial assemblages. These microorganisms originate from diverse sources, including soil, air, and nearby plants, and are driven by the plant and environmental parameters [19]. Despite the open nature of the phyllosphere and its exposure to various environmental factors, recent research indicates the presence of a core set of leaf-associated microbiota that persist in the phyllosphere, with plant genotype having a substantial impact on its microbiome [20, 21]. Moreover, leaf metabolites can influence the composition and function of phyllosphere microorganisms. These microorganisms, in turn, can affect the production and release of leaf metabolites through their interactions with the plant host. However, there is limited experimental evidence on leaf metabolites and their impact on the phyllosphere microbiota, particularly concerning the extent of plant genotype influence on the quantity and quality of leaf metabolites and their relationship with the resistomes [12, 16, 17]. Understanding the genetic effects on the phyllosphere-specific microbiome and its interactions with metabolites is essential for predicting and monitoring plant resistomes throughout plant evolution.

Wheat, a staple food crop that feeds approximately 20% of the world’s population, has undergone extensive evolutionary processes, including natural hybridization, polyploidization, domestication, and mutation over more than 10 000 years [22]. Given that alterations in microorganisms can induce changes in antibiotic resistance gene (ARG) profiles, we hypothesized that specific genetic variations among wheat genotypes lead to distinct leaf metabolites, which in turn develop a specialized phyllosphere microbiome that influences the abundance and distribution of ARGs. Our objective was to identify the key wheat metabolites involved in this process and to elucidate the mechanisms by which these metabolites affect the microbial community and its resistomes. To test this hypothesis, we selected 16 wheat cultivars to (i) characterize the genetic impacts on the abundance and diversity of ARGs using high-throughput quantitative PCR (HT-qPCR); (ii) estimate the mobility and risks of the phyllosphere resistomes by profiling mobile genetic elements (MGEs) and virulence factor genes (VFGs); (iii) illustrate variations in phyllosphere microbial communities across different wheat cultivars; (iv) identify key microbial taxa contributing to differences in the phyllosphere resistomes; and (v) comprehensively elucidate interactions between plant-specific metabolites, ARGs, and key taxa through nontargeted metabolic analysis, metadata analysis, and confirmatory experiments.

## Materials and methods

### Experiment design and sample collection

**Figure 1:**
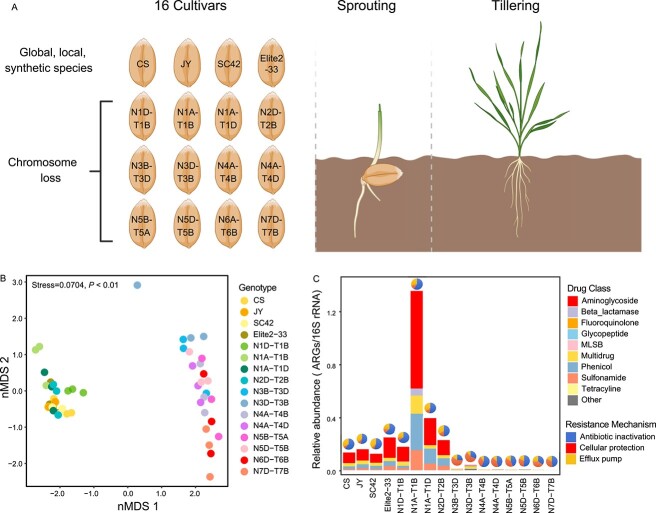
Effect of wheat genotype on phyllosphere resistome. **A** Graphical representation of the experimental design, involving 16 wheat cultivars. **B** Nonmetric multidimensional scaling analysis based on the Bray–Curtis distances showing the distinct distribution patterns of phyllosphere ARG profiles across different wheat genotypes. **C** Relative abundance (copies per 16S rRNA gene copy) of ARGs in various wheat genotypes.

A pot experiment was conducted to explore the relationships between wheat genotypes and phyllosphere resistomes. Experimental soil (0–20 cm depth) was collected from farmland in Ningbo, Zhejiang Province, China, and air-dried in the laboratory at 25°C for several days. Subsequently, the dried soil was sieved through a 2-mm nylon mesh and moistened with sterile water to achieve 60% of the maximum field capacity. To stabilize and enhance the activity of the native soil microbial communities and provide a consistent baseline for subsequent experimental treatments, the soil was preincubated at 25°C in the dark for 2 weeks to activate soil microorganisms. We analyzed the genetic variations within the three subgenomes of wheat, designated as A, B, and D. This nomenclature is based on the evolutionary origins of modern hexaploid wheat (*Triticum aestivum*), which comprises three distinct sets of chromosomes from different ancestral species: *Triticum urartu* (A subgenome), a species related to *Aegilops speltoides* (B subgenome), and *Aegilops tauschii* (D subgenome). Each subgenome contributes a set of chromosomes, resulting in a total of 21 chromosome pairs (7 pairs per subgenome), labeled as 1A to 7A for the A subgenome, 1B to 7B for the B subgenome, and 1D to 7D for the D subgenome. These genomes harbor unique sets of genes and genetic variations, contributing to wheat’s wide genetic diversity and adaptability to various environments. Therefore, sixteen wheat cultivars obtained from the Jiangsu Academy of Agricultural Sciences (Jiangsu Province, China), including those widely distributed worldwide (Chinese spring) [23], local species from northern and southern China, synthetic hexaploid wheat, and cultivars with missing chromosomes, were used for the microcosm experiment (Fig. 1A and Table S1).

Prior to setting up the experiment, seeds of the 16 wheat cultivars were sterilized in 30% H_2_O_2_ for 15 min, followed by thorough washing with sterilized MilliQ water. Subsequently, the seeds were germinated on moist filter paper for 48 h at 28°C in the dark. The germinated seeds were then sown in PVC pots (16.8 cm × 13 cm × 15.8 cm) containing 3 kg of preincubated soil, with each pot receiving five seeds. Two weeks after sowing, the seedlings were thinned to 3 per pot. The wheat seedlings were grown in a 12-hour light (light intensity 10 000 lux) and 12-hour dark cycle, with 65% relative humidity, maintaining a daytime temperature of 28°C and a nighttime temperature of 22°C. Each wheat cultivar was allocated three pots, which were re-randomized daily during the growth period. Soil moisture content was regularly adjusted to 35% by weight using deionized water. After 7 weeks of growth, corresponding to the tillering stage of wheat development, the wheat leaves were collected and stored at −80°C for further analysis. The tillering stage is characterized by an increased leaf surface area and heightened microbial interactions, creating a nutrient-rich environment conducive to microbial growth and horizontal gene transfer, which may pose a risk for ARG transmission.

### DNA extraction

DNA was extracted from the wheat phyllosphere using the FastDNA Spin Kit for soil (MP Biomedical, Santa Ana, California, USA). Briefly, 5 g of leaf tissue was sonicated and shaken in a 250-mL conical flask containing 100 mL of 0.01 M phosphate-buffered saline (PBS). The mixture was then filtered first through a sterilized nylon net and then through a cellulose membrane with a pore size of 0.22 μm. The filters were subsequently cut into pieces using sterilized pair of scissors, and DNA was extracted according to the manufacturer’s protocol [24]. DNA concentration and quality were determined using a NanoDrop 2000 (Thermo Fisher, USA) and gel electrophoresis with a 1.0% agarose gel, respectively. DNA extracts were stored at −20°C until further analysis.

### High-throughput quantitative PCR

The ARG patterns in the phyllosphere samples were analyzed using high-throughput quantitative PCR (HT-qPCR) with the Wafergen SmartChip Real-time PCR system (Wafergen Inc., USA) [25]. A total of 384 primer sets (refer to Table S2) were employed, comprising 319 primer sets targeting nearly all major classes of ARGs, 7 taxonomic genes, 57 primer sets targeting MGEs (including 9 insertional genes, 11 plasmid genes, 10 transposase genes, 3 integron-integrase genes, and 24 other MGEs), along with primer sets for 16S rRNA genes. The 384 primer sets employed in this study were carefully selected based on previously published studies to ensure specificity and efficiency [25, 26]. In addition, these primer sets have been previously employed in various studies across different habitats, including soil [25], water [27], phyllosphere [28], and animal guts [29], demonstrating their reliability and reproducibility. The PCR amplification protocol involved an initial step of enzyme activation at 95°C for 10 min, followed by 40 cycles. Each cycle consisted of denaturation at 95°C for 30 s and annealing at 60°C for 30 s. Melt curves were automatically generated using Wafergen software. A threshold cycle (Ct) of 31 was employed as the detection limit in the present study. The relative copy number of ARGs and MGEs was calculated using the formula: relative gene copy number = 10^(31 − Ct)(10/3)^, Ct represents the threshold cycle obtained during PCR amplification.

### High-throughput sequencing

To characterize the bacterial and fungal communities in the wheat phyllosphere across different genotypes, specific bacterial (515F/806R for the V4 region of 16S rRNA gene) [30] and fungal (gITS7/ITS4ngs for the ITS2 region) [31] primer sets with barcodes were selected for amplification. The amplified products were submitted to Majorbio Bio-pharm Technology Co., Ltd (Shanghai, China) for high-throughput sequencing using the MiSeq System (Illumina; 300 cycles) platform. Microbiome bioinformatics analyses were conducted using QIIME 2 [32]. Raw sequencing data were initially demultiplexed and quality-filtered using the q2-demux plugin, followed by denoising with DADA2 [33]. All amplicon sequence variants (ASVs) were aligned using multiple alignment with fast Fourier transform via q2-alignment [34], and a phylogenetic tree was constructed with FastTree [35]. Taxonomic assignments for bacterial and fungal ASVs were performed using the q2-feature-classifier [36] with the classify-sklearn Naive Bayes taxonomy classifier via 99% comparisons to the SILVA 138 [37] and UNITE 8.0 [38] reference databases, respectively. Moreover, our study exclusively targeted bacteria and fungi, sequences corresponding to mitochondria, archaea, chloroplasts, and unassigned reads for 16S rRNA gene were removed before downstream analysis.

### Nontarget metabolomics analysis

Leaf tissues were washed with sterilized 1 × PBS, and 50 mg of each sample was extracted using a methanol solution (4:1, v/v) containing an internal standard. The mixture was processed using a tissue crusher and ultrasound, followed by protein precipitation. Following centrifugation, supernatants were transferred for LC–MS/MS analysis using a UHPLC-Q Exactive HF-system (Thermo Fisher Scientific). Quality control samples were used to monitor the stability of the analysis. Data processing steps included noise removal, database searching, and normalization. Detailed procedures are provided in the supporting information (refer to S1 text).

### Metagenome analysis

To further reveal the hosts of the wheat phyllosphere resistomes, 15 samples, including genotypes JY, Elite2–33, N1A-T1D, N3B-T3D, and N5D-T5B were selected for metagenomic analysis using the DNBSEQ-T7 instrument. Data analysis was processed on the Majorbio platform (http://www.majorbio.com). The paired-end Illumina reads were trimmed of adaptors, and low-quality reads (length < 50 bp or with a quality value <20 or having N bases) were removed by fastp (version 0.20.0) [39]. The reads were then assembled with MEGAHIT (version 1.1.2) to create contigs of ≥300 bp [40].Gene prediction was conducted using MetaGene [41], and open reading frames (ORFs) ≥100 bp were translated using the NCBI translation table (http://www.ncbi.nlm.nih.gov/Taxonomy/taxonomyhome.html/index.cgi?chapter=tgencodes#SG1). CD-HIT (version 4.6.1) was used to construct a nonredundant gene catalog with 90% sequence identity and coverage [42]. Quality-controlled reads were mapped to the nonredundant gene catalog with 95% identity using SOAP aligner (version 2.21) [43] to evaluate gene abundance. Representative sequences were aligned to the NCBI NR database using DIAMOND (version 0.8.35) with an e-value cutoff of 1e-5 for taxonomic annotations [44]. The predicted gene protein sequences were compared with the Kyoto Encyclopedia of Genes and Genomes (KEGG) databases to obtain functional annotation information. Antibiotic-resistant annotation was conducted using the ARGs online analysis pipeline (ARGs-OAP) [45, 46]. Assessing high-risk ARGs is crucial, as they pose a significant threat to public health. In the present study, the health risk of ARGs to humans was divided into four degrees (Q1, Q2, Q3, and Q4) based on human accessibility, mobility, pathogenicity, and clinical availability [47]. Virulence factors were annotated using DIAMOND (version 0.8.35) against the VFDB database (http://www.mgc.ac.cn/VFs/) with an e-value cutoff of 1e-5 [44].

### Validation microcosm setup

To simulate interactions between specialized metabolites and microorganisms, we selected an accessible, relatively important, and referable upregulated leaf metabolite DIMBOA-Glu (Toronto Research Chemicals, CAS: 113565–32-5). The objective was to explore the influence of exogenous DIMBOA-Glu on *Pseudomonas* species. Four treatments were designed: 0 ppb (without DIMBOA-Glu addition), 10 ppb (10 μg DIMBOA-Glu per kg soil), 100 ppb (100 μg DIMBOA-Glu per kg soil), and 1000 ppb (1000 μg DIMBOA-Glu per kg soil). These treatments were conducted in 100-mL glass beaker microcosms, with each treatment allocated in triplicate. All microcosms were maintained under conditions consistent with those of the wheat development experiment. After four weeks, soil samples were collected for DNA extraction. The 16S rRNA gene amplicon sequencing was conducted to profile the microbial communities, and the abundance of *Pseudomonas* spp. was quantified using quantitative PCR.

### Statistical analysis

Data analysis was performed using Microsoft Excel 2020 for calculating averages and sums. Microbial diversity was assessed by calculating the Shannon index for α-diversity, whereas β-diversity was estimated using the Bray–Curtis distance between samples. The nonmetric multidimensional scaling analysis (nMDS) and significance tests (PERMANOVA test) based on Bray–Curtis distance were employed to evaluate differences in ARGs and phyllosphere microbial communities among different wheat cultivars, respectively, using the “vegan” [48] and “labdsv” [49] packages. Linear discriminant analysis effect size (LEfSe) was calculated with the Kruskall–Wallis test (*P* < 0.05). “igraph” package was used to construct the co-occurrence network based on Spearman’s correlation matrix (|*r*| > 0.7, *P* < 0.01), and then visualized with Gephi 0.10 version. Heatmaps were generated using the “vegan” package [48] in R4.3.1. Principal coordinate analysis (PCA) plot was generated from Bray–Curtis similarity matrices using “ggplot2” in R4.3.1 to distinguish the metabolite profiles in different wheat genotypes. Volcano analysis and the variable importance in the projection (VIP) values from orthogonal projections to latent structures discriminant analysis (OPLS-DA) were further used to distinguish the leaf metabolites associated with high-ARG and low-ARG wheat genotypes. Canonical correlation analysis (CCA) was conducted with the “vegan” package in R 4.3.1 to investigate the correlations between the phyllosphere resistomes and microbiome (bacterial community, fungal community), MGEs, and metabolites. We used the value of nMDS axis 1 to represent the bacterial and fungal β-diversity, and PCA axis 1 to represent the overall pattern of leaf metabolome. All bar charts, bubble plots, scatter diagrams, and OLS regressions in this study were generated using the “ggplot2” package in R 4.3.1 [50], with significance considered at *P* < 0.05.

## Results

### Abundance and diversity of ARGs in the phyllosphere

A total of 104 ARGs were observed in the wheat phyllosphere samples, covering 12 major classes of antibiotics commonly administered to humans and animals, such as aminoglycoside, beta-lactams, and tetracycline, among others (Fig. S1A). The nMDS analysis and PERMANOVA revealed that the phyllosphere resistomes formed two major clusters (Adonis, *P* < 0.01, Bray–Curtis distance) (Fig. 1B). For instance, higher abundances and diversity of phyllosphere ARGs were found in the wheat genotypes of CS, JY, SC42, Elite2–33, N1D-T1B, N1A-T1D, N1A-T1B, and N2D-T2B compared to other genotypes such as N3B-T3D, N3D-T3B, N4A-T4B, N4A-T4D, N5B-T5A, N5D-T5B, N6D-T6B, and N7D-T7B (Figs 1C and S1A). According to ARG abundances, the phyllosphere samples were classified into high- and low-ARG abundance genotype groups. Furthermore, considering resistance mechanisms*,* antibiotic inactivation was the dominant mechanism in all phyllosphere samples. However, the proportions of antibiotic inactivation and efflux pump mechanisms were higher in phyllosphere samples with high-ARG abundances, whereas phyllosphere ARGs in wheat genotypes with low abundance exhibited higher proportions of cellular protection mechanisms (Fig. 1C).

### Mobility and risks of phyllosphere resistome

**Figure 2:**
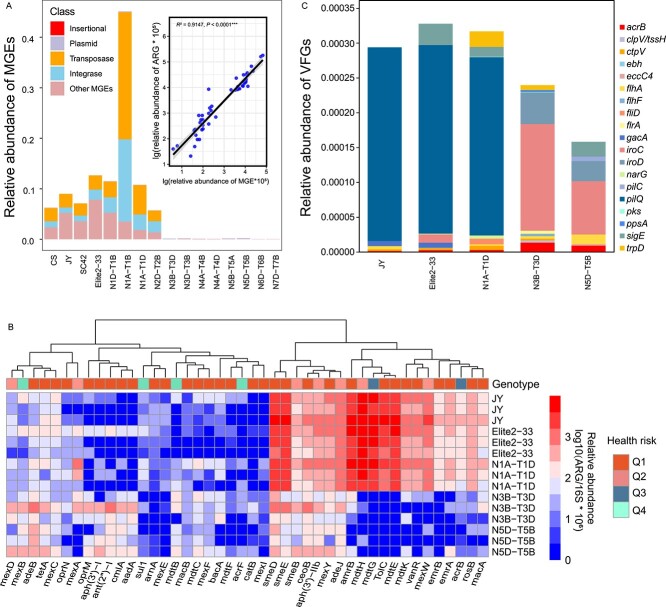
Effects of wheat genotype on mobility and risks of the phyllosphere resistome. **A** Relative abundance of mobile genetic elements (MGEs) in different wheat genotypes, small panel showing the correlation between the relative abundance of ARGs and MGEs. **B** Metagenomic analysis revealing the relative abundance of virulence factor genes (VFGs) across various wheat genotypes. **C** Metagenomic analysis revealing the relative abundance of the high-risk ARGs in the wheat genotypes JY, Elite2–33, N1A-T1D, N3B-T3D, and N5D-N5B.

Thirty-nine MGEs (including insertional genes, transposase genes, integrase genes, and plasmids) were targeted in this study, with transposase genes significantly enriched in phyllospheres with higher ARG abundances (ranging from 0.026 to 0.252 copies per 16S rRNA genes) compared to low-ARG genotypes (ranging from 0 to 0.021 copies per 16S rRNA genes) (Figs 2A and S1B). OLS regression analysis further revealed a significant correlation between the relative abundance of ARGs and MGEs (*R*^2^ = 0.9147, *P* < 0.0001) (Fig. 2A).

To further corroborate the distinct ARG patterns and assess risks, phyllosphere metagenomes were generated from five wheat genotypes, three with high-ARG abundances (JY, Elite2–33, and N1A-T1D) and two with low abundances (N3B-T3D and N5D-T5B). Consistent with HT-qPCR results, genotypes JY, Elite2–33, and N1A-T1D contained higher ARG abundances than N3B-T3D and N5D-T5B (Fig. S2). Most detected ARGs in the wheat phyllosphere belonged to Q1, the highest-risk ARGs, and conferred multidrug resistance (Fig. 2B). Additionally, 19 VFGs were detected in the wheat phyllosphere, with genotypes harboring high abundances of ARGs containing a higher total abundance of VFGs (Fig. 2C). Pathogenic bacteria in high-ARG phyllospheres carried more abundant *pilQ* genes, whereas *iroC* and *iroD* were dominant VFGs in low-ARG phyllospheres (Fig. 2C).

### Phyllosphere microbiome varied with wheat genotype

**Figure 3:**
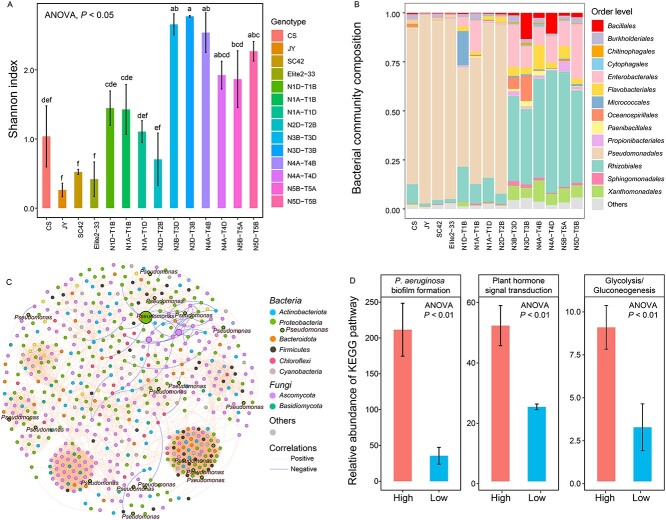
Responses of phyllosphere microbial communities in wheat to different genotypes. **A** Alpha-diversity of phyllosphere bacterial communities estimated by the Shannon index. **B** Composition of phyllosphere bacterial communities classified at the order level. **C** Co-occurrence network illustrating interactions between bacterial taxa and fungal taxa in the phyllosphere with high-ARG abundance. **D** Comparison of the abundances of KEGG pathways related to *Pseudomonas aeruginosa* biofilm formation, plant hormone signal transduction, and glycolysis/gluconeogenesis between high-ARG (high) and low-ARG (low) phyllosphere samples.

Both bacterial and fungal α-diversity in the wheat phyllosphere significantly varied among plant genotypes (ANOVA, *P* < 0.05) (Figs 3A and S3A). For example, the Shannon index of bacterial diversity in genotypes JY, Elite2–33, and N1A-T1D was significantly lower than in N3B-T3D and N5D-T5B (ANOVA, *P* < 0.05) (Fig. 3A). Nonmetric multidimensional scaling analysis of Bray–Curtis distances demonstrated that variations in wheat genotypes were the primary drivers of bacterial and fungal β-diversity in the wheat phyllosphere (Adonis, *P* < 0.01) (Figs S3B and S4). At the order level, most bacterial communities in high-ARG abundance phyllospheres belonged to *Pseudomonadales* (ranging from 50.7% to 96.1%)*,* whereas *Rhizobiales* were abundant in the low-ARG phyllospheres (Fig. 3B). Moreover, the majority of fungal sequences were classified as *Ascomycota* (Fig. S3C). The co-occurrence network indicated strong interactions between *Pseudomonas* species, other bacterial taxa, and fungal taxa (Fig. 3C).

To explore the impact of wheat genetic variation on phyllosphere microbial function, we analyzed differences in KEGG metabolic capacities between high-ARG and low-ARG phyllospheres. Ninety KEGG orthologs (KOs) showed distinct distribution patterns between high-ARG and low-ARG groups (Fig. S5), with significant enrichment of KOs involved in various pathways such as *Pseudomonas aeruginosa* biofilm formation*,* plant hormone signal transduction*,* and glycolysis/gluconeogenesis, in high-ARG phyllospheres (ANOVA, *P* < 0.01) (Fig. 3D).

### *Pseudomonas* spp. enrichment in high-ARG phyllospheres

LEfSe analysis indicated that bacteria *Pseudomonas* spp. were significantly enriched in high-ARG phyllospheres (Fig. S6). The co-occurrence networks of bacteria and fungi differed significantly between the two groups of phyllosphere samples, with *Pseudomonas* spp. playing a central role in the network of high-ARG phyllospheres (Figs 3C and S7).

**Figure 4:**
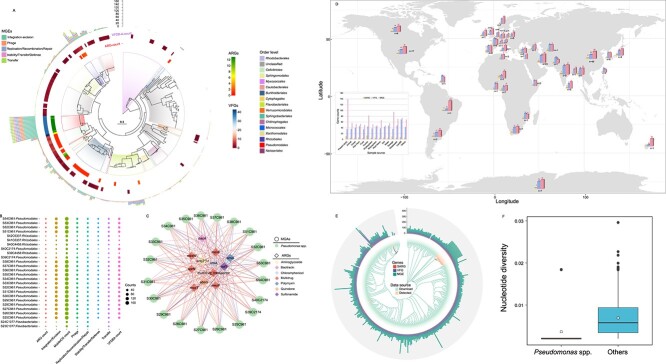
Distribution of ARGs, MGEs, and VFGs in metagenome-assembled genomes (MAGs) of *Pseudomonas* species. **A** Maximum-likelihood phylogenetic tree showing the phylogenetic distributions of ARGs, MGEs, and VFGs on 472 MAGs derived from phyllosphere samples. **B** Bubble plot depicting the number of ARGs, MGEs, and VFGs associated with multidrug resistant bacterial taxa (ARG > 2). **C** Network analysis revealing the co-occurrence patterns between MAGs belonging to *Pseudomonas* spp. and various ARG subtypes. **D** Geographic distribution of samples, displaying the number of ARGs, MGEs, and VFGs in *Pseudomonas* spp. from various habitats. **E** Maximum-likelihood phylogenetic tree showing the phylogenetic distributions of ARGs, MGEs, and VFGs on *Pseudomonas* spp. genome from the present studied phyllosphere samples and those downloaded from NCBI database. **F** A comparison of median genome-wide nucleotide diversity between *Pseudomonas* spp. and other taxa.

A total of 472 MAGs were recovered from the phyllosphere samples, and functionally annotated for ARGs, VFGs, and MGEs. These MAGs covered 16 bacterial orders, with 165 MAGs remaining unclassified. In addition, the MAGs from *Pseudomonadales* occupied a large proportion in the high-ARG phyllospheres (Fig. S8). Among these, 22 MAGs from *Pseudomonas* spp. were identified, most representing multidrug-resistant bacteria carrying numerous VFGs and MGEs (Figs 4A and B). The 12 ARGs captured on *Pseudomonas* spp. encoded resistance to various antibiotics, including aminoglycoside, bacitracin, chloramphenicol, polymyxin, quinolone, sulfonamide, and multidrug. In addition, ten of these detected ARGs are considered high-risk (Fig. 4C). Ten MAGs from the order *Rhizobiales* also carried ARGs (ranging from 0 to 2) and VFGs (ranging from 2 to 6), but the number of ARGs within *Rhizobiales* was ten times lower compared to *Pseudomonas* spp. (Fig. 4A). To validate that *Pseudomonas* spp. are more important carriers of ARGs compared to other species, 350 *Pseudomonas* spp. genomes (Fig. 4D) and 100 *Rhizobiales* genomes (Table S3) were downloaded from NCBI database, covering diverse hosts and habitats. Reassembly and annotation of these genomes revealed that *Pseudomonas* spp. are high-capacity ARG-carriers, with each genome containing an average of 3–17 ARGs, multiple VFGs (ranging from 23 to 79), and MGEs (ranging from 31 to 89) worldwide (Fig. 4D). *Pseudomonas* spp. detected in this study contained a higher number of MGEs compared to those downloaded from the NCBI (Fig. 4E). Moreover, *Pseudomonas* spp. exhibited significantly lower nucleotide diversity than other species (*P* < 0.001) (Fig. 4F). In contrast, *Rhizobiales* genomes revealed that *Rhizobiales* is a low-capacity ARG carrier, with only 20% of taxa containing ARGs (ranging from 1 to 4) (Table S3).

### Specific metabolites induced the enrichment of *pseudomonas* spp. in phyllosphere

**Figure 5:**
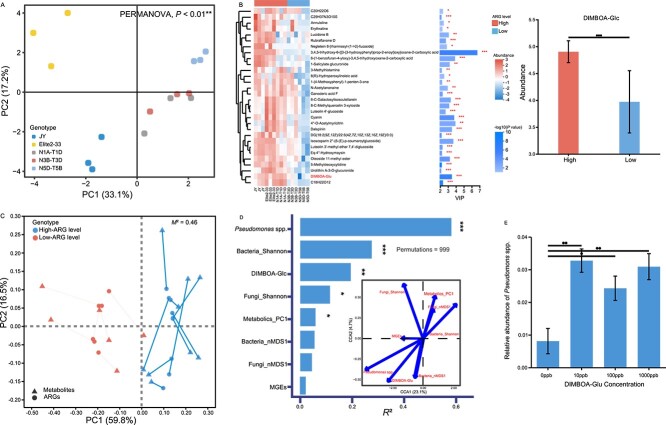
Responses of leaf metabolic profiles to plant genetic variation and their relationship with the phyllosphere resistome. **A** Principal component analysis (PCA) showing the effect of wheat genotype on the leaf metabolome. **B** Heatmap and variable important in projection (VIP) value bar chart illustrating the expression patterns of upregulated metabolites in each leaf sample. The log2FC represents the fold change in metabolite expression between two groups, and the log10(*P* value) indicates the significance of expression differences, with higher values denoting more significant differences. Each dot corresponds to a specific metabolite, and dot size reflects VIP values. **C** Procrustes analysis of ARG profiles with leaf metabolites in high-ARG level and low-ARG level samples. **D** Canonical correspondence analysis (CCA) depicting the quantitative correlation between phyllosphere ARG patterns and key factors. **E** Validation microcosm experiment showing the influence of DIMBOA-Glu concentrations on the relative abundance of *Pseudomonas* species.

Metabolomics analysis revealed that leaf metabolic profiles were highly distinct among different wheat cultivars (Adonis, *P* < 0.01) (Fig. 5A). Furthermore, we used a volcano plot (Fig. S9) and VIP values (Fig. 5B) to investigate biomarker metabolites in the high-ARG wheat. The results showed that 101 metabolites were significantly enriched in the wheat leaf tissue of high-ARG genotypes (ANOVA, *P* < 0.05) (Fig. S9). Most of these metabolites belonged to organooxygen compounds, prenol lipids, and carboxylic acids and derivatives. These results showed that DIMBOA-Glc is one of most important chemicals driven the divergence between high-ARG wheat and low-ARG wheat, with the abundance of DIMBOA-Glc in the leaves of JY, Elite2–33, and N1A-T1D being significantly higher compared to N5D-T5B (Log2FC > 1, *P* < 0.001) (Figs 5B and S10).

The procrustes analysis indicated a significant relationship between leaf metabolites and phyllosphere resistomes (Fig. 5C). CCA analysis further showed that the first two axes explained 27.8% of the variance between the selected variables. And *Pseudomonas* spp. were the most important drivers of the plant resistomes (Fig. 5D). Furthermore, our analysis revealed strong correlations between multiple upregulated metabolites and the abundance of ARGs, and microorganisms enriched in the high-ARG phyllosphere (Figs S11 and S12). Additionally, the presence of DIMBOA-Glc showed significant positive correlations with both the total abundance of phyllosphere *Pseudomonas* spp. (Spearman’s *r* = 0.79, *P* < 0.01) (Fig. S11) and ARGs (Spearman’s *r* = 0.76, *P* < 0.01) (Fig. S12).

Compared to the overall metabolome patterns, DIMBOA-Glu exhibited a stronger relationship with the phyllosphere resistome (Fig. 5D). Furthermore, to corroborate the hypothesis that specific metabolites induce the enrichment of *Pseudomonas* spp. in the high-ARG phyllosphere, a microcosm experiment was conducted to explore the effects of exogenous DIMBOA-Glu on the *Pseudomonas* species. The results showed that the addition of DIMBOA-Glu significantly altered the overall patterns of bacterial communities (Adonis, *P* < 0.05) (Fig. S13). At the phylum level, exogenous DIMBOA-Glu increased the relative abundance of *Actinobacteria* whereas decreasing that of *Firmicutes* (Fig. S13). At the genus level, the relative abundances of *Pseudomonas* spp. were significantly enhanced in the microbial communities with the addition of DIMBOA-Glu, regardless of concentration of DIMBOA-Glu (ANOVA, *P* < 0.05) (Fig. 5E). Conversely, an opposite trend was observed in the absolute abundance of *Pseudomonas* species (Fig. S14).

## Discussion

**Figure 6:**
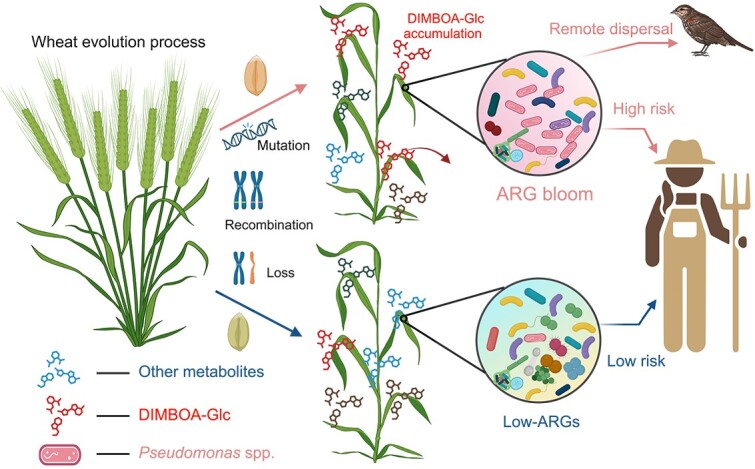
Proposed model for genetic-dependent, DIMBOA-Glu, and *Pseudomonas*-mediated ARG accumulation in the wheat phyllosphere (Created with BioRender.com).

Understanding how genetic variation in plants, including chromosome mutations, losses, and gains, impacts the antibiotic resistome of its phyllosphere is crucial for developing sustainable agricultural practices. This study investigated the associations between genetic variation in wheat and its antibiotic resistome within the specific context of chromosomal changes. Our results demonstrated that host genetic variation significantly regulates the phyllosphere antibiotic-resistance gene (ARG) profiles of globally distributed and important staple crops such as wheat. Wheat cultivars exhibited high abundances and diversities of ARGs, accompanied by the proliferation of *Pseudomonas* species, which are widely distributed carriers of multiple ARGs, as evidenced by metadata. Additionally, we found that plant genetic variation reshaped metabolic profiles, and changes in the concentration of specific metabolites were associated with the abundances of ARGs and *Pseudomonas*. Therefore, we suggest that plant genotype has a considerable impact on the establishment of the phyllosphere microbiome by producing distinct metabolic profiles. These profiles, in turn, profoundly increase the relative abundance of *Pseudomonas* spp*.*, thereby enhancing the resistomes. Our experimental validation confirmed that the leaf metabolite DIMBOA-Glu significantly altered bacterial communities and increased the relative abundance of *Pseudomonas* species (Fig. 6). These findings elucidate how plant genetic information regulate phyllosphere resistome, and provide insights into the potential impacts of natural and artificial plant evolution on the occurrence and dissemination of antibiotic resistance.

In this study, we observed that specialized genotypes harbor higher levels of ARGs, mobile genetic elements (MGEs), and virulence factor genes (VFGs) in their phyllosphere, indicating that the host genome contributes to shaping the phyllosphere resistome and affecting the risks of ARGs. For example, we found that the loss of chromosomes 1A, 1B, and 1D, as well as the duplication of chromosome 1B, significantly enriched the abundance and diversity of ARGs. Consequently, we propose that manipulating group-1 chromosomes may promote the accumulation of ARGs in the wheat phyllosphere. Furthermore, such genetic variation increases the abundance of multidrug resistance genes, which exhibit the highest average risk index [47]. Previous research indicates that bacteria in stressed environments are more likely to evolve and maintain multidrug resistance genes. For instance, in nutrient-limited or antimicrobial-exposed settings, multidrug resistance offers a significant competitive advantage, allowing bacteria to survive and proliferate despite the adverse conditions [51, 52]. Therefore, genetic variation, such as chromosome loss or gene mutations in wheat, can lead to physiological alterations. These alterations may affect the availability of essential nutrients or metabolites in the phyllosphere, creating a selective environment that favors the growth of specific microbial populations, including those with high-risk ARGs. The presence and accumulation of high-risk ARGs in the wheat phyllosphere microbiome can potentially transfer to pathogenic bacteria, leading to the emergence of multidrug-resistant infections [53]. Moreover, the integration of wheat and wheat-derived products into the food chain can facilitate the spread of these ARGs [54, 55], emphasizing the need to consider the increased risk of antibiotic resistance to the ecosystem during the domestication and/or genetic engineering breeding processes.

Microbial communities are well-known drivers of the antibiotic resistome [1, 25, 56]. During plant evolution, resident microorganisms co-evolve with their hosts and adapt to them, significantly altering plant microbial communities, which in turn reshapes the resistome [57]. For example, wheat genotypes with high-ARG abundances in the phyllosphere are accompanied by an elevated population of *Pseudomonas* species. Previous studies suggest that *Pseudomonas* spp. can colonize a broad spectrum of habitats due to their ability to exploit diverse nutritional sources and adapt to new environmental conditions [58, 59]. The significant variation in *Pseudomonas* spp. abundances across different samples, despite all wheat genotypes being grown in the same glasshouse with a common source of air microbiomes, suggests that genetic variations among the wheat genotypes are influence the microbial community composition. Specific genotypic traits might lead to differences in metabolite composition, thereby creating niches that favor the proliferation of specific bacterial orders like *Pseudomonadales* in certain genotypes. Meanwhile, abundant VFGs and MGEs reside in these multidrug-resistant *Pseudomonas* species. In particular, the abundances of KOs involved in *P. aeruginosa* biofilm formation significantly enriched in the phyllosphere samples with higher abundances of ARGs. *P. aeruginosa* possesses a highly conserved core genome with low-sequence diversity and a highly variable accessory genome that communicates with other *Pseudomonas* spp. and genera via horizontal gene transfer [60]. This partly explains the high abundances of ARGs and MGEs in high-ARG wheat cultivars, whereas the lower prevalence of *Pseudomonas* spp. in low-ARG phyllospheres explains the low abundances of ARGs in low-ARG wheat cultivars. Moreover, compared to overall phyllosphere microbial communities, the abundance of *Pseudomonas* spp. plays a more important role in shaping the phyllosphere resistome. We further queried 350 *Pseudomonas* spp. genomes and 100 *Rhizobiales* genomes from the global dataset covering a variety of habitats to assess the presence of ARGs. All strains of *Pseudomonas* spp. host a wide spectrum of environmental ARGs, MGEs, and VFGs, yet *Rhizobiales* is a low-capacity ARG carrier, with only 20% of taxa containing ARGs (ranging from one to four). Overall, these findings suggest that although the capacity to carry ARGs is common among various bacterial orders, the degree of enrichment can vary significantly. The differential abundance of ARGs and associated genetic elements in *Pseudomonas* spp. versus *Rhizobiales* highlights the complex interactions between host plant genetics and microbial community composition. These results underscore the importance of considering the specific microbial taxa when evaluating the resistome of plant-associated microbiomes. These findings indicate that host genetic variation influences microbial community composition and resistome dynamics in a taxon-specific manner, and the bloom of *Pseudomonas* spp. in the wheat phyllosphere is the main contributor to antibiotic resistance. In other words, we suggest that *Pseudomonas* spp. can be considered a reliable environmental predictor for ARG accumulation in wheat phyllospheres during wheat breeding.

Distinct leaf metabolic profiles were exhibited in high-ARG wheat cultivars and low-ARG wheat cultivars. Moreover, multiple upregulated metabolites in high-ARG wheat cultivars showed a strong correlation with various phyllosphere ARGs. Thus, leaf metabolites are probably a functional determinant of phyllosphere ARG patterns. Our study further suggests that the upregulated metabolite DIMBOA-Glc enriches *Pseudomonas* species*,* thereby enhancing phyllosphere ARGs. In general, plants synthesize and release specialized metabolites into their environment, serving as chemical cues for recruiting and shaping microbial colonizers [15, 61, 62]. DIMBOA-Glc is one of the most frequently identified benzoxazinoids in wheat leaves, with its concentration varying widely among different wheat varieties. Previous studies have reported that benzoxazinoids could regulate rhizosphere microbial communities and even attract root *Pseudomonas* spp. in maize [14, 63, 64]. Therefore, the positive and strong relationships between *Pseudomonas* spp. and DIMBOA-Glc indicate that the enrichment of DIMBOA-Glc leads to a more specialized community capable of resisting or degrading benzoxazinoid compounds enriched in the phyllosphere. This is supported by the enrichment of plant hormone signal transduction and glycolysis/gluconeogenesis potential in phyllosphere microorganisms. Recent study demonstrated that plant-derived benzoxazinoids could act as antibiotics [14]. Thus, benzoxazinoids may kill specific bacteria and exert selection pressure on other microorganisms, which in turn increases the abundances of ARGs. *Pseudomonas* spp. contain the highest proportion of regulatory genes observed within a bacterial genome, including a substantial number of genes dedicated to the catabolism, transport, and efflux of organic compounds. Additionally, *Pseudomonas* spp. possess numerous potential chemotaxis systems. These genetic features reflect an evolutionary adaptation that enables *Pseudomonas* spp. to thrive in diverse environments and resist a variety of antimicrobial substances [59]. This is evidenced by our observation that antibiotic inactivation and efflux pumps are the dominant resistance mechanisms in the phyllosphere of wheat cultivars with high-ARG and DIMBOA-Glc abundance. Moreover, we experimentally demonstrated that the release of DIMBOA-Glc significantly enhances the relative abundances of *Pseudomonas* spp. whereas decreasing their absolute abundance*.* These findings validate the antimicrobial effects of DIMBOA-Glc on microbial communities [14, 64]. In addition to direct antimicrobial activities, a previous study reported that DIMBOA could act as a chemoattractant for *Pseudomonas putida* KT2440 [64], reinforcing our observation that DIMBOA-Glc drives the accumulation of *Pseudomonas* spp. in the phyllosphere. Overall, the changes of leaf metabolites caused by genetic variation over the course of evolution may lead to a highly specialized microbial community that could enrich phyllosphere ARGs.

The plant microbiome serves as a critical interface between human and natural microbiomes, representing a pivotal pathway for human exposure to environmental antibiotic resistance [65]. Thus, ARG carriers selected by certain plant metabolites during evolutionary processes may exacerbate the dispersal of antibiotic resistance through the food chain, direct contact, and globalization, posing significant risks to human health [66]. Although our research focused on wheat, recent studies have highlighted the significant impact of plant genetic variation on the microbiome across various crops, including maize, tomato, and soybean [4, 17, 67]. For example, a recent study observed that root exudate purine or its derivatives enrich root-associated *Pseudomonas* spp. and improve wild soybean growth under salt stress [67]. These studies provide insights into the generalizability of our observed interactions between host genetics, microbial communities, and ARGs.

In summary, our study unveiled the significant role of host genotypic variation in shaping the patterns of ARG in the wheat phyllosphere. By integrating data on the phyllosphere microbiome, metabolic profiles, and global *Pseudomonas* spp. genome data, along with findings from validation experiments on metabolites and bacteria, we demonstrated that chemically distinct leaf metabolites resulting from host genetic variation can lead to the development of a highly specialized microbial community capable of enriching phyllosphere ARGs. Our study addressed fundamental questions regarding the factors influencing the phyllosphere resistomes throughout plant evolution. Further research is warranted to elucidate the molecular mechanisms underlying the interactions between wheat genetic variations and the microbiome. Longitudinal studies across different growth stages and environmental conditions will provide insights into the stability and evolution of these interactions. Our findings can inform breeding programs aimed at developing crop varieties with reduced potential for ARG accumulation. By selecting genotypes with lower ARG prevalence, we can mitigate the spread of antibiotic resistance in agricultural settings. Additionally, strategies such as the targeted application of beneficial microorganisms or amendments that modulate plant–microbe interactions can manage the microbiome composition, thereby reducing high-risk ARGs.

## Supplementary Material

Supporting_information_wrae144

## Data Availability

All of the raw sequences have been deposited in the National Center for Biotechnology Information (NCBI) Sequence Read Archive (SRA) under Accession No. PRJNA1097066.

## Additional information

Extended data are available for this paper.
