# Supplementary material for: Host genetic variation and specialized metabolites from wheat leaves enriches for phyllosphere Pseudomonas spp. with enriched antibiotic resistomes
Source: ISME J. 2024 Jul 29;18(1):wrae144. doi: 10.1093/ismejo/wrae144 (PMC11334211; doi:10.1093/ismejo/wrae144)
Supplement: Supporting_information_wrae144 [file supporting_information_wrae144.docx]

**Supporting information for**

**Host genetic variation and specialized metabolites from wheat leaves enriches for phyllosphere *Pseudomonas* spp. with enriched antibiotic resistomes**

Qiang Xiang ^a,b^, Da Lin ^a,b,c^, Zai-Jun Yang ^d^, Rui-Xia Han ^a,b^, Tian-Lun Zhang ^a,b,c^, Qing-Lin Chen ^a,b^, Dong Zhu ^a,b,*^, Josep Penuelas ^e,f^ , Yong-Guan Zhu ^a,g^

^a^ Key Laboratory of Urban Environment and Health, Ningbo Urban Environment Observation and Research Station, Institute of Urban Environment, Chinese Academy of Sciences, Xiamen 361021, China.

^b^ Zhejiang Key Laboratory of Urban Environmental Processes and Pollution Control, CAS Haixi Industrial Technology Innovation Center in Beilun, Ningbo 315830, China.

^c^ University of Chinese Academy of Sciences, Beijing 100049, China.

^d^ Key Laboratory of Southwest China Wildlife Resources Conservation (ministry of education), College of Life Science, China West Normal University, Nanchong, Sichuan 637009, China.

^e^ CSIC, Global Ecology Unit CREAF-CSIC-UAB, Bellaterra, Barcelona 08193, Catalonia, Spain

^f^ CREAF, Campus Universitat Autònoma de Barcelona, Cerdanyola del Vallès, Barcelona 08193, Catalonia, Spain

^g^ State Key Laboratory of Urban and Regional Ecology, Research Center for Eco-Environmental Sciences, Chinese Academy of Sciences, Beijing, 100085, China.

^*^ Corresponding Author: Dong Zhu: [dzhu@iue.ac.cn](mailto:dzhu@iue.ac.cn)

**Contents**

**S1 text：**Non-target metabolomics analysis

**Figures**

**Figure S1** Effect of wheat genotype on the ARG and MGE diversity. Number of **A** ARG and **B** MGEs detected in the phyllosphere samples across different wheat genotypes.

**Figure S2** Metagenomic analysis reveals the relative abundance of antibiotic resistance genes (ARGs) in different genotype wheats.

**Figure S3** Response of phyllosphere fungal communities in wheat to different genotypes. **A** The phyllosphere fungal alpha-diversity estimated by Shannon index. **B** Non-metric multidimensional scaling analysis (nMDS) based on the Bray-Curtis distances showing the distinct distribution patterns of phyllosphere fungal profiles in different wheat genotypes. **C** The composition of phyllosphere fungal communities classified at the phylum level.

**Figure S4** Non-metric multidimensional scaling analysis based on the Bray-Curtis distances showing the distinct distribution patterns of phyllosphere bacterial profiles in different wheat genotypes.

**Figure S5** Heatmap shows the abundances of KEGG pathways that exhibited significant variations between high-ARG and low-ARG phyllosphere samples.

**Figure S6** Cladograms generated by LEfSe indicating enrichment of bacterial taxa between high-level and low-level ARG abundances. The central yellow dot in each cladogram represents kingdom; each successive circle is one step lower phylogenetically (phylum, class, order, family, and genus). Red and green on the bacterial trees indicate the taxa that are enriched in phyllosphere with high- ARG and low-ARG level abundances, respectively.

**Figure S7** The co-occurrence network between bacterial taxa and fungi taxa in the phyllosphere with low-ARG abundance level.

**Figure S8** The relative abundance of metagenome-assembled genomes (MAGs) derived from phyllosphere.

**Figure S9** Volcanic maps show the metabolites with large and statistically significant variations between high-ARG and low-ARG phyllosphere samples.

**Figure S10** The average abundance of DIMBOA-Glu in different genotypes. * 0.01 < *P* ≤ 0.05, ** 0.001 < *P* ≤ 0.01, *** *P* ≤ 0.001.

**Figure S11** Heatmap shows the correlation between leaf metabolites and enriched bacterial taxa in phyllosphere.

**Figure S12** Heatmap shows the correlation between leaf metabolites and different classes ARGs in phyllosphere.

**Figure S13** Principal coordinate analysis (PCoA) based on Bray−Curtis distances depicting the distinct distribution patterns of bacterial communities. Circos graph showing the microbial compositions in different DIMBOA-Glu concentrations.

**Figure S14** Microcosm experiment shows the influence of DIMBOA-Glu concentrations on the absolute abundance of *Pseudomonas* species.

**Tables**

**Table S1** The description of selected wheat cultivars

**Table S2** Information of 384 genes primers

**Table S3** The number of ARGs and VFGs located on Rhizobiales

**Total:**

**Number of figures: 14**

**Number of tables: 3**

**S1 text：Non-target metabolomics analysis**

Prior to metabolomic analysis, leaf tissues were washed with sterilized 1× PBS to remove soil and dust. Each leaf sample (50 mg) was weighed precisely and metabolites were extracted using a 400 µL methanol (4:1, v/v) solution containing 0.02 mg/mL L-2-chlorophenylalanine as an internal standard. Extraction involved incubation at -10°C, followed by homogenization using a High-throughput tissue crusher (Wonbio-96c) at 50 Hz for 6 minutes and subsequent ultrasonic treatment at 40 kHz for 30 minutes at 5°C. Protein precipitation was achieved by storing samples at -20°C for 30 minutes, followed by centrifugation at 13,000 g and 4°C for 15 minutes. Supernatants were collected and stored in sample vials for LC-MS/MS analysis.

For quality control, a pooled sample was prepared by combining equal volumes of all samples. This quality control sample underwent the same processing and analysis procedure as other samples and was injected at regular intervals (every 12 samples) to monitor system stability. LC-MS/MS analysis was performed using a UHPLC-Q Exactive HF system (Thermo Fisher Scientific). Raw data were processed using Progenesis QI software (Waters Corporation). Internal standard peaks and known false positive peaks (e.g., noise, column bleed) were removed from the data matrix. Metabolite identification utilized the Human Metabolome Database (HMDB), Metlin, and the Majorbio Database. Processed data were uploaded to the Majorbio cloud platform for further analysis. Metabolic features present in at least 80% of any sample set were retained. For samples with metabolite levels below the lower limit of quantitation, minimum values were imputed, and each metabolic feature was normalized by sum to minimize variability. The response intensity of mass spectrum peaks was normalized using the sum normalization method to obtain a consistent data matrix. Variables with a relative standard deviation (RSD) greater than 30% in quality control samples were excluded. Finally, data were log-transformed to prepare the final data matrix for subsequent analysis.

**Chromatographic conditions:** Samples (2 µL) were separated using a HSS T3 column (100 mm × 2.1 mm i.d., 1.8 µm) and analyzed by mass spectrometry. Mobile phases consisted of 0.1% formic acid in water (95:5, v/v) (solvent A) and 0.1% formic acid in acetonitrile:isopropanol (47.5:47.5:5, v/v) (solvent B). The solvent gradient was programmed as follows: 0% B to 24.5% B from 0 to 3.5 min (0.4 mL/min); 24.5% B to 65% B from 3.5 to 5 min (0.4 mL/min); 65% B to 100% B from 5 to 5.5 min (0.4 mL/min); 100% B (0.4 mL/min) to 100% B (0.6 mL/min) from 5.5 to 7.4 min; 100% B to 51.5% B from 7.4 to 7.6 min (0.6 mL/min); 51.5% B to 0% B from 7.6 to 7.8 min (0.5 mL/min to 0.4 mL/min); and 0% B to 0% B from 7.8 to 9 min (0.4 mL/min). The column temperature was maintained at 40°C, and samples were stored at 4°C during analysis.

**Mass spectrometric conditions:** Mass spectrometric data were acquired using a Thermo UHPLC-Q Exactive HF-X Mass Spectrometer with an electrospray ionization (ESI) source operating in positive or negative ion mode. Conditions included a heater temperature of 425°C, capillary temperature of 325°C, sheath gas flow rate of 50 arb, auxiliary gas flow rate of 13 arb, ion-spray voltage floating at -3500V in negative mode and 3500V in positive mode, and normalized collision energy set at 20-40-60V for MS/MS. Full MS resolution was 60,000 and MS/MS resolution was 7,500. Data-dependent acquisition (DDA) mode covered a mass range of 70-1050 m/z.

**Data preprocessing and annotation:** After completing mass spectrometry detection, the raw LC/MS data was processed using Progenesis QI software (Waters Corporation, Milford, USA). This software generated a three-dimensional data matrix in CSV format containing sample information, metabolite names, and mass spectral response intensities. During preprocessing, internal standard peaks and known false positive peaks (including noise, column bleed, and derivatized reagent peaks) were systematically removed from the data matrix. The data were then dereplicated and peak pooled to enhance accuracy and reliability. Metabolites were identified using the Human Metabolome Database (HMDB) (http://www.hmdb.ca/), Metlin (https://metlin.scripps.edu/), and the Majorbio Database. Subsequently, the processed data were uploaded to the Majorbio cloud platform (https://cloud.majorbio.com) for further analysis. Metabolic features detected in at least 80% of any sample set were retained. For samples where metabolite levels fell below the lower limit of quantitation, minimum metabolite values were imputed. Each metabolic feature was normalized by sum to minimize errors due to sample preparation and instrument instability. Variables with a relative standard deviation (RSD) > 30% of quality control (QC) samples were removed, and a log10 transformation was applied to achieve a final normalized data matrix for subsequent analysis.

**Differential metabolites analysis:** The matrix file obtained after data preprocessing underwent variance analysis. Using the R package ropls (Version 1.6.2), principal component analysis (PCA) and orthogonal partial least squares discriminant analysis (OPLS-DA) were performed. The model's stability was evaluated using 7-fold cross-validation. Student's t-test and fold difference analysis were conducted to identify significantly different metabolites. Significantly different metabolites were selected based on the Variable Importance in Projection (VIP) scores from the OPLS-DA model and the p-value from Student’s t-test. Metabolites with VIP > 1 and *P* < 0.05 were considered statistically significant. Identified differential metabolites between groups were then annotated and mapped to biochemical pathways using database searches (e.g., KEGG, http://www.genome.jp/kegg/). Metabolites were categorized based on the pathways they are involved in or the functions they perform. Enrichment analysis was performed to determine statistically significantly enriched pathways using Fisher’s exact test implemented in scipy.stats Python packages (https://docs.scipy.org/doc/scipy/). This analysis helped elucidate the functional roles and biochemical pathways associated with the identified differential metabolites.

**Figure S1**


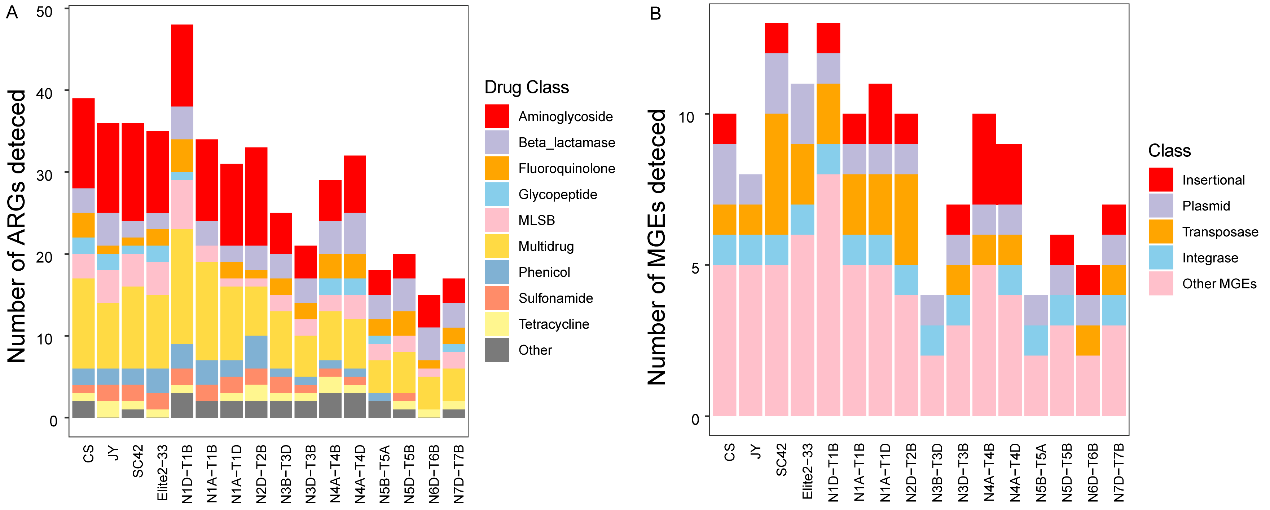


Figure S1 Effect of wheat genotype on the ARG and MGE diversity. Number of **A** ARG and **B** MGEs detected in the phyllosphere samples across different wheat genotypes.

**Figure S2**


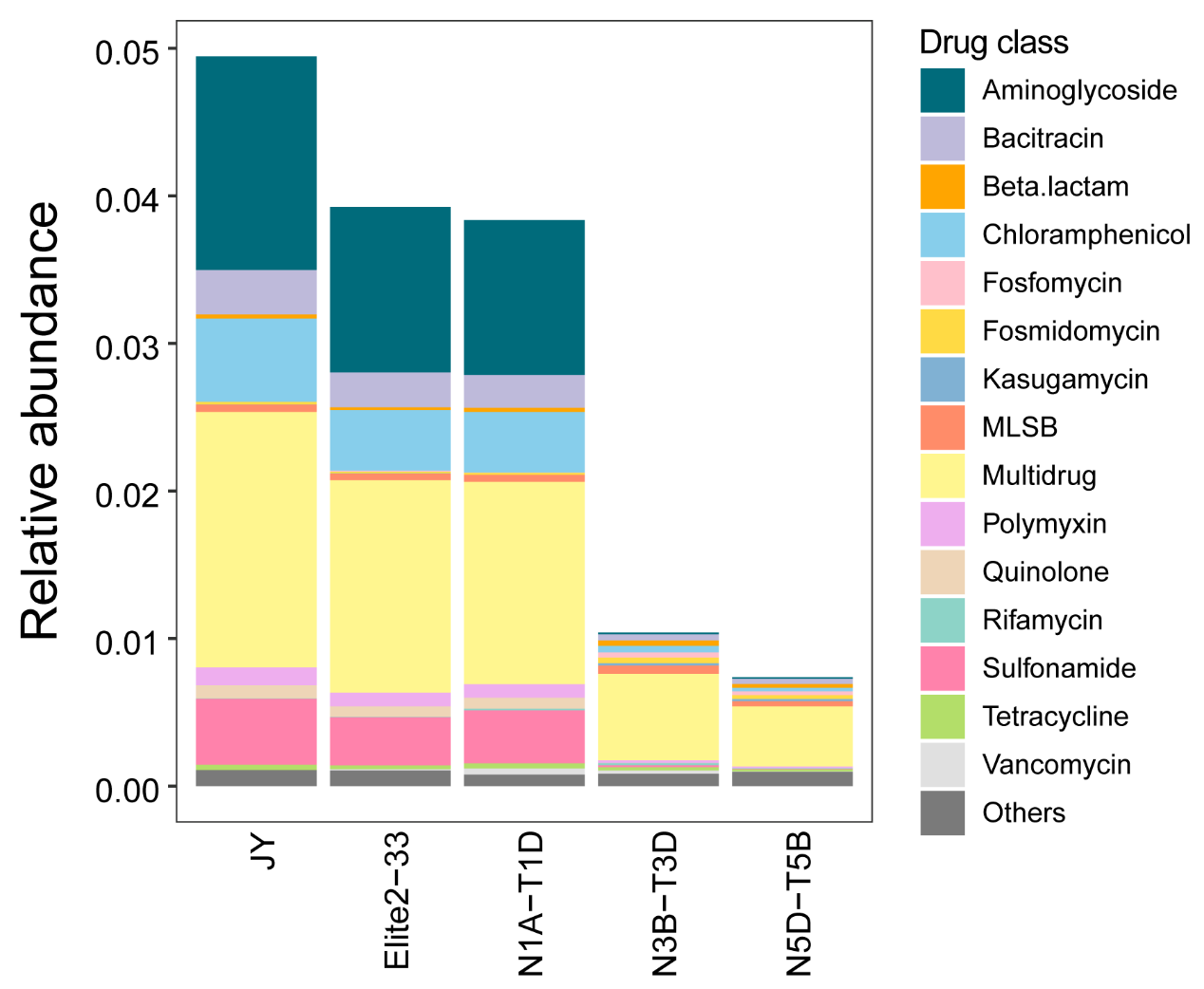


Figure S2 Metagenomic analysis reveals the relative abundance of antibiotic resistance genes (ARGs) in different genotype wheats.

**Figure S3**


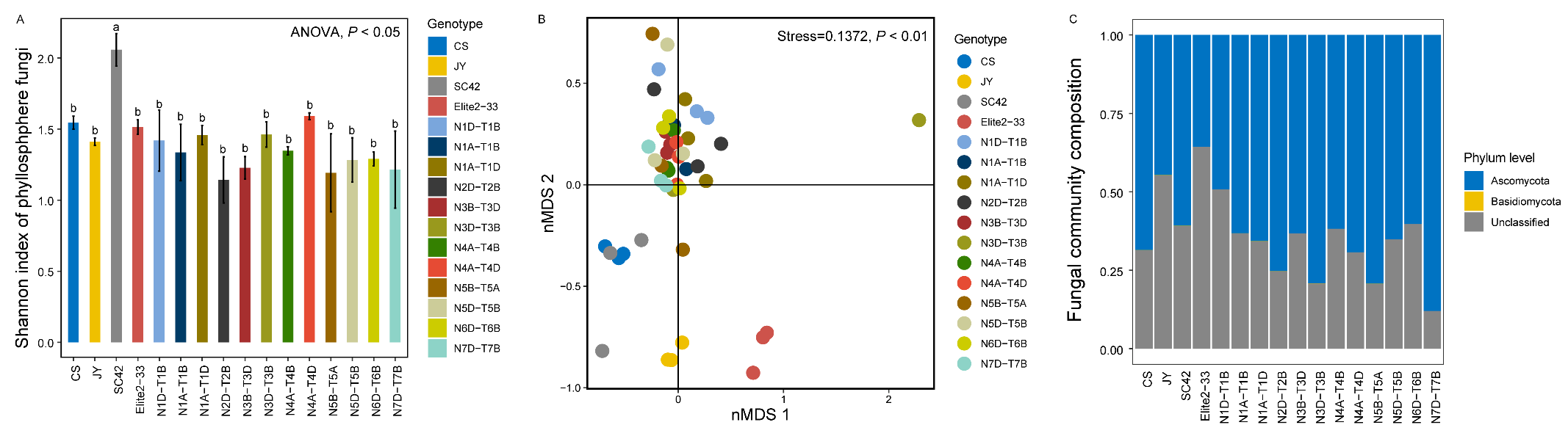


Figure S3 Response of phyllosphere fungal communities in wheat to different genotypes. **A** The phyllosphere fungal alpha-diversity estimated by Shannon index. **B** Non-metric multidimensional scaling analysis (nMDS) based on the Bray-Curtis distances showing the distinct distribution patterns of phyllosphere fungal profiles in different wheat genotypes. **C** The composition of phyllosphere fungal communities classified at the phylum level.

**Figure S4**


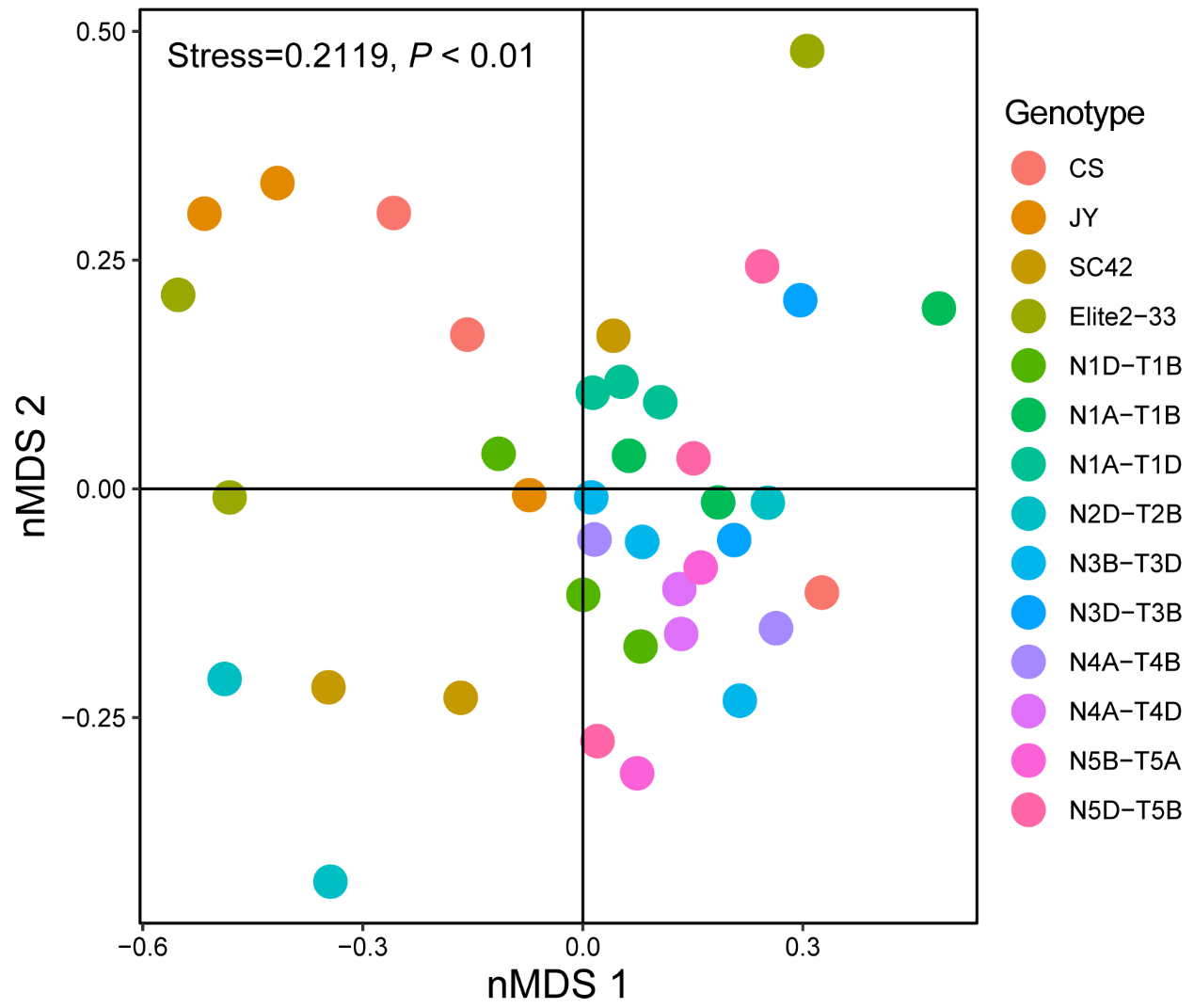


Figure S4 Non-metric multidimensional scaling analysis based on the Bray-Curtis distances showing the distinct distribution patterns of phyllosphere bacterial profiles in different wheat genotypes.

**Figure S5**

**
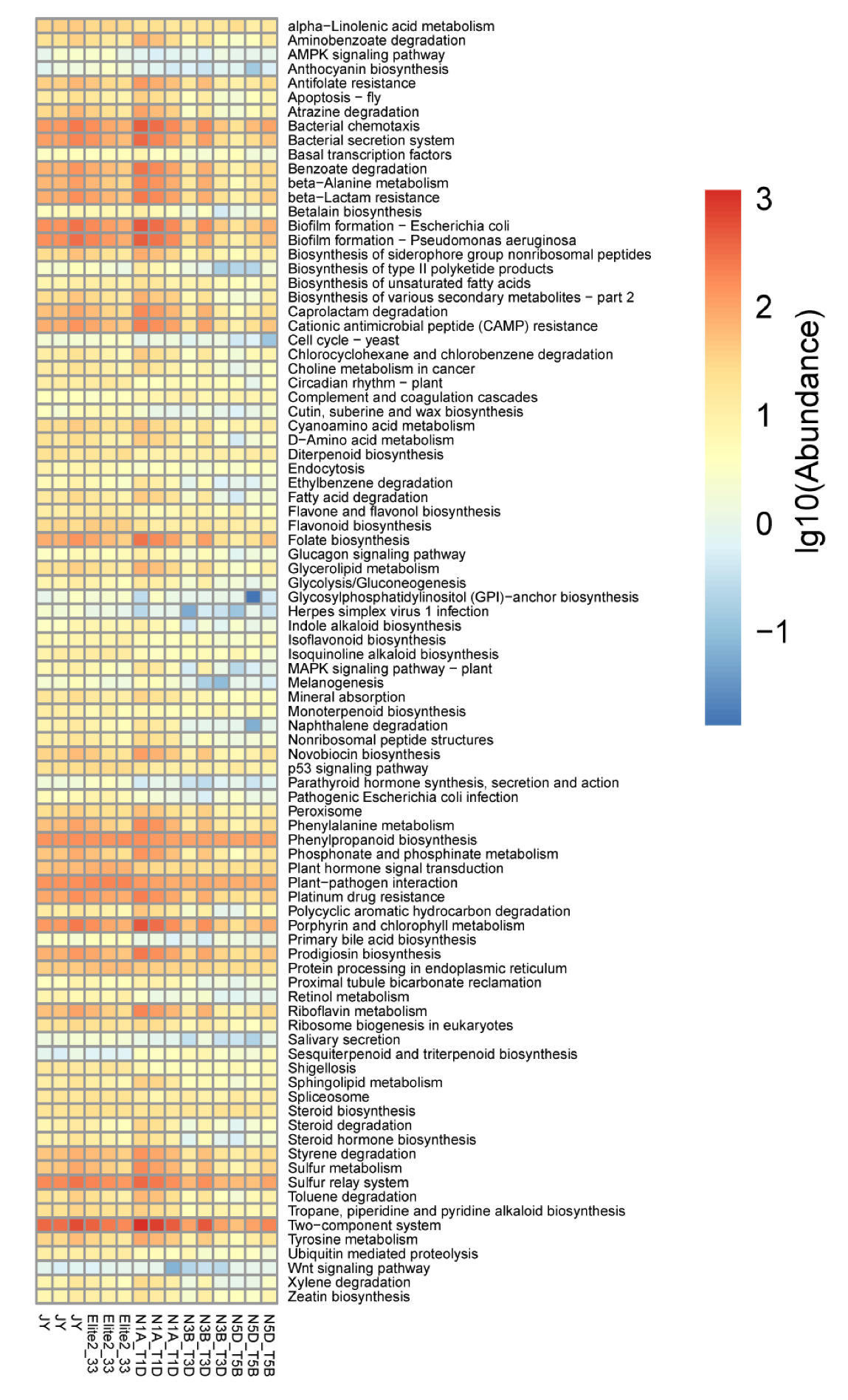
**

Figure S5 Heatmap shows the abundances of KEGG pathways that exhibited significant variations between high-ARG and low-ARG phyllosphere samples.

**Figure S6**


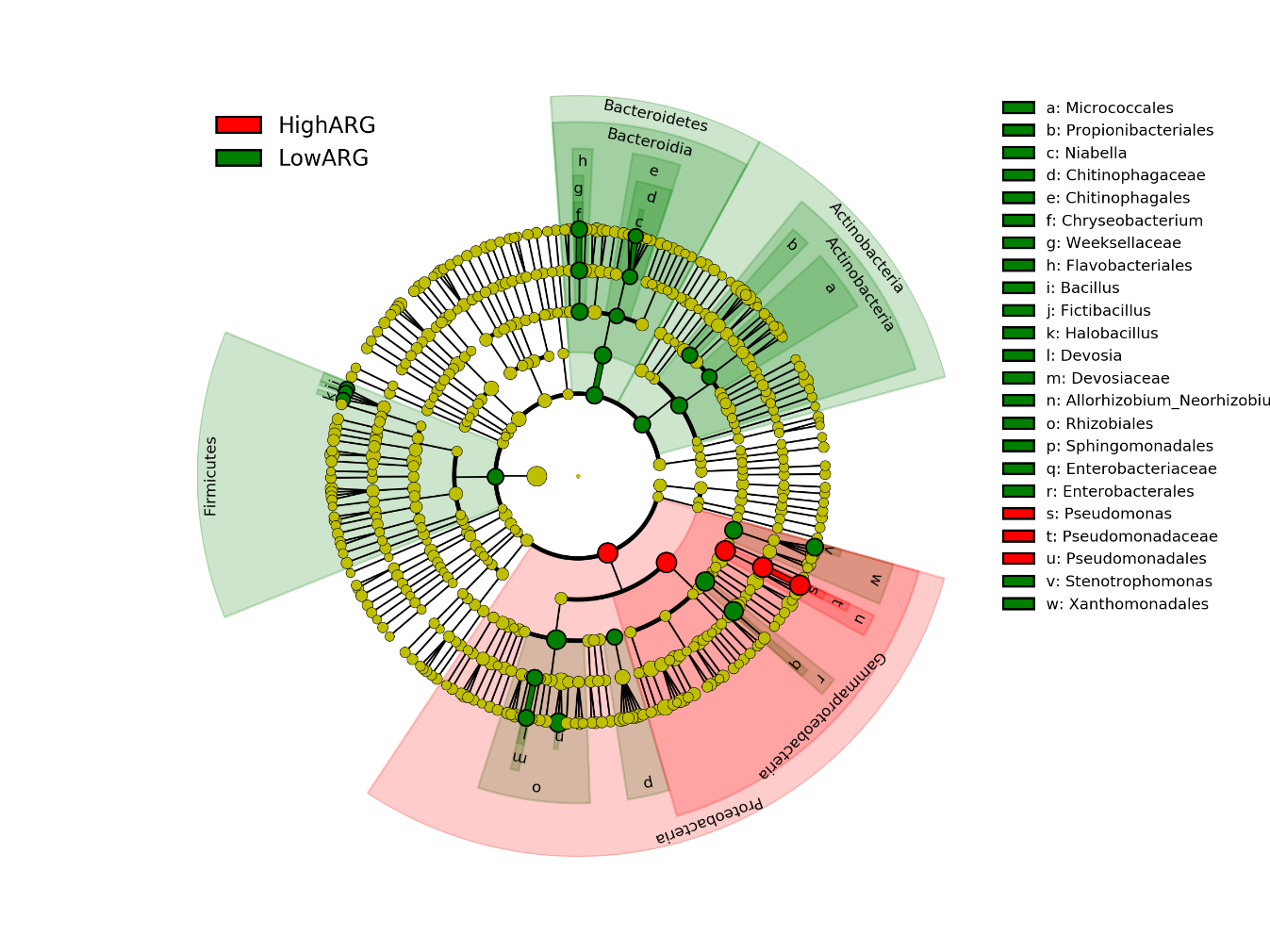


Figure S6 Cladograms generated by LEfSe indicating enrichment of bacterial taxa between high-level and low-level ARG abundances. The central yellow dot in each cladogram represents kingdom; each successive circle is one step lower phylogenetically (phylum, class, order, family, and genus). Red and green on the bacterial trees indicate the taxa that are enriched in phyllosphere with high- ARG and low-ARG level abundances, respectively.

Figure S7


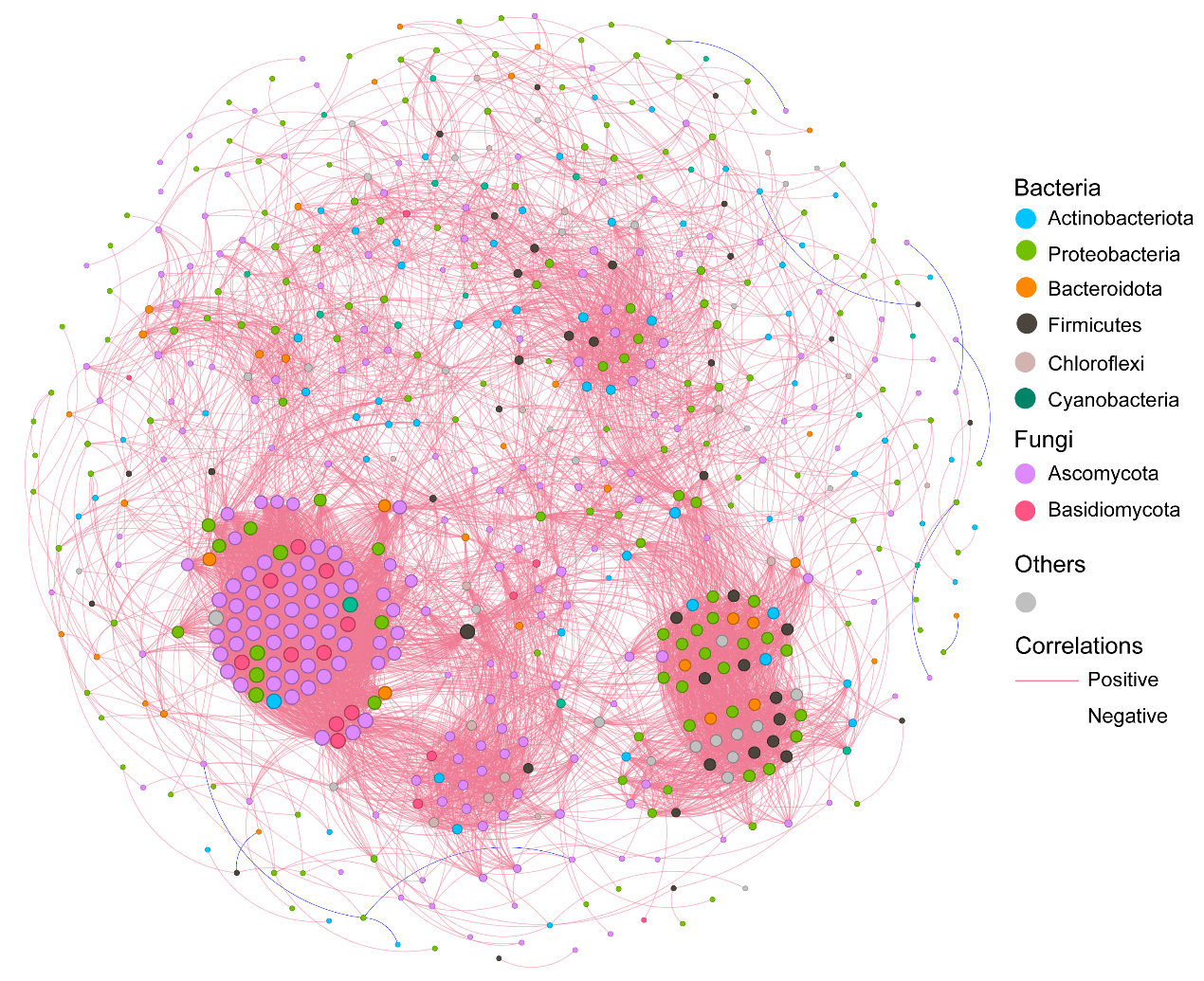


Figure S7 The co-occurrence network between bacterial taxa and fungi taxa in the phyllosphere with low-ARG abundance level.

**Figure S8**


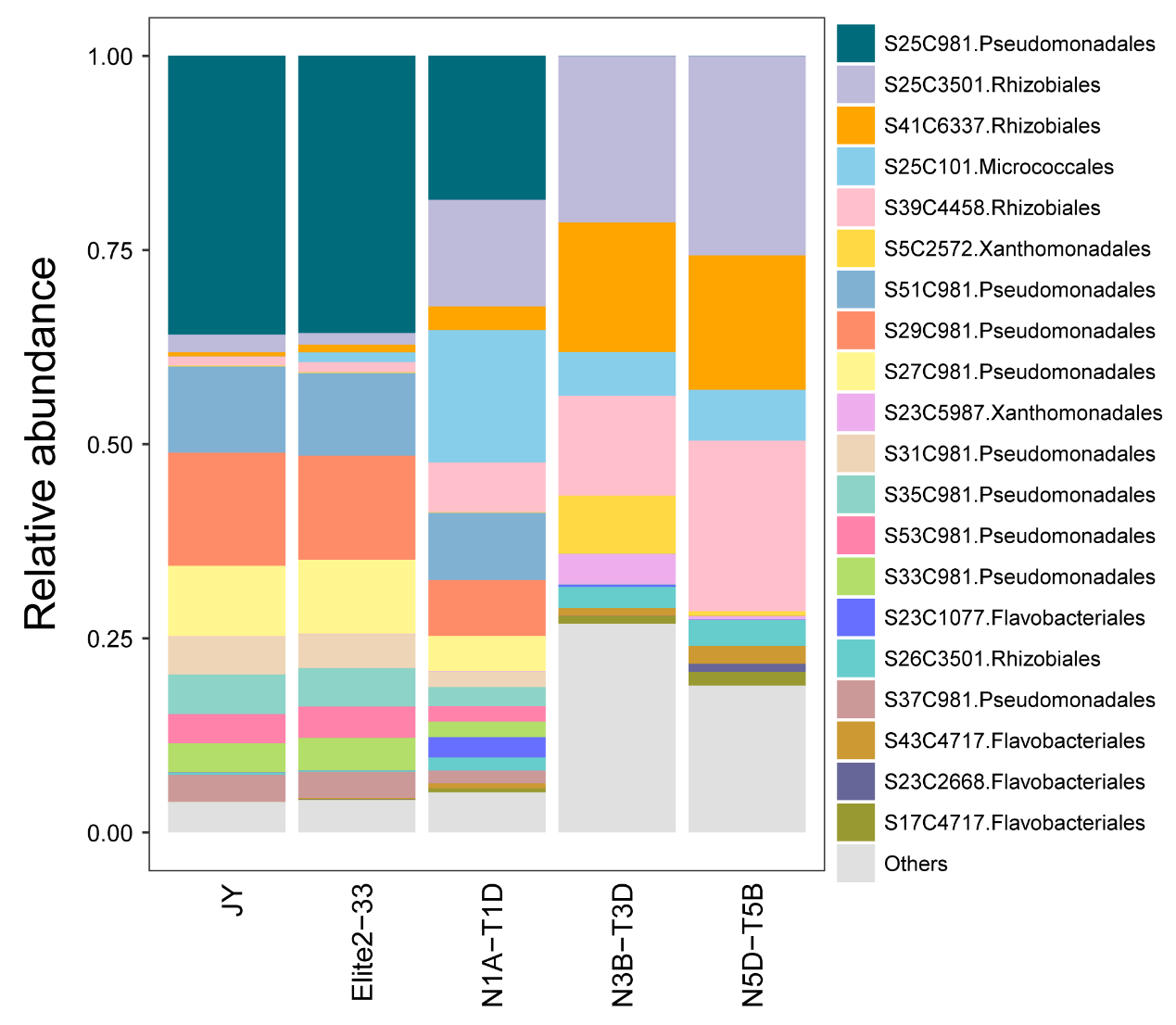


Figure S8 The relative abundance of metagenome-assembled genomes (MAGs) derived from phyllosphere.

**Figure S9**


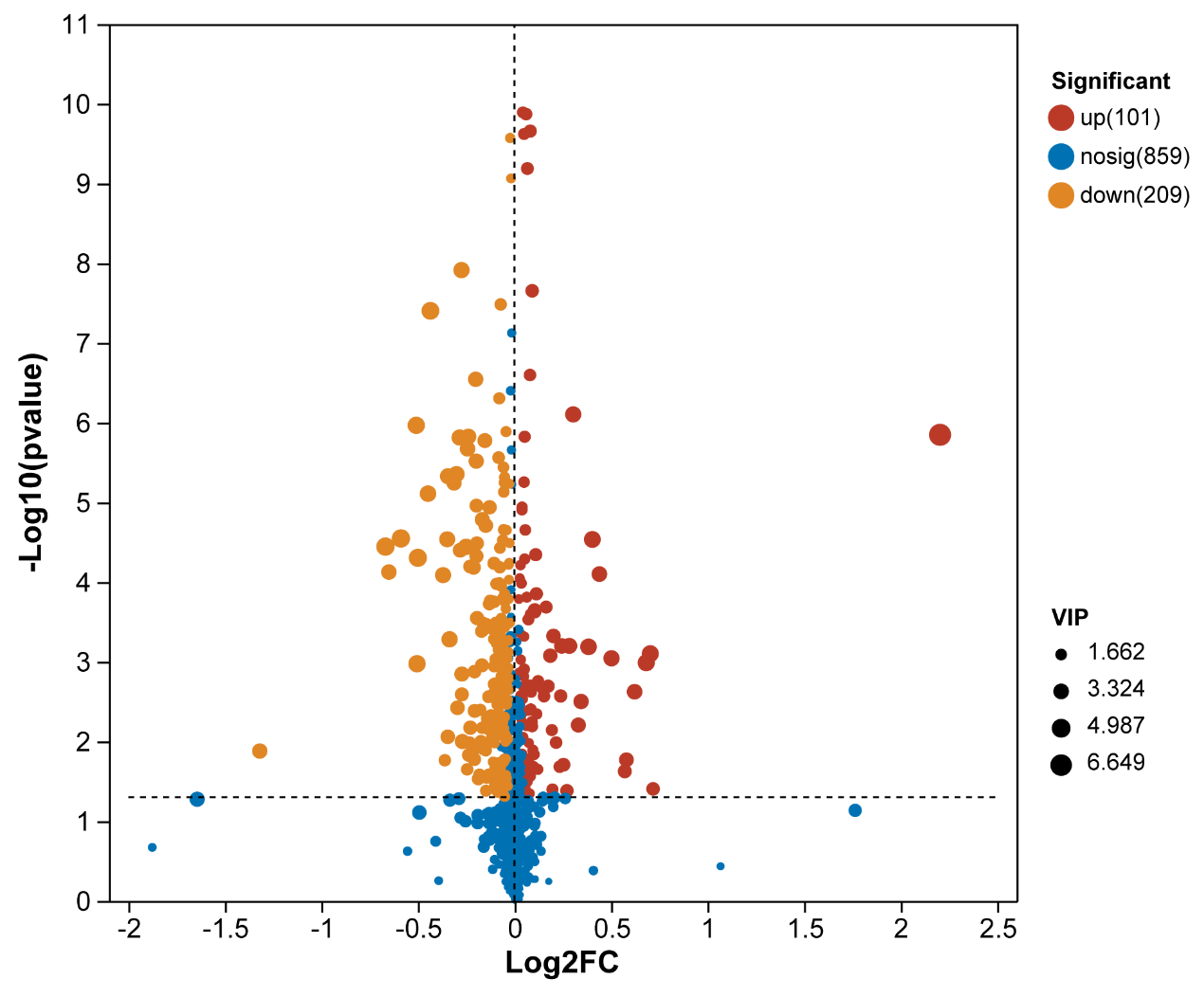


Figure S9 Volcanic maps show the metabolites with large and statistically significant variations between high-ARG and low-ARG phyllosphere samples.

**Figure S10**


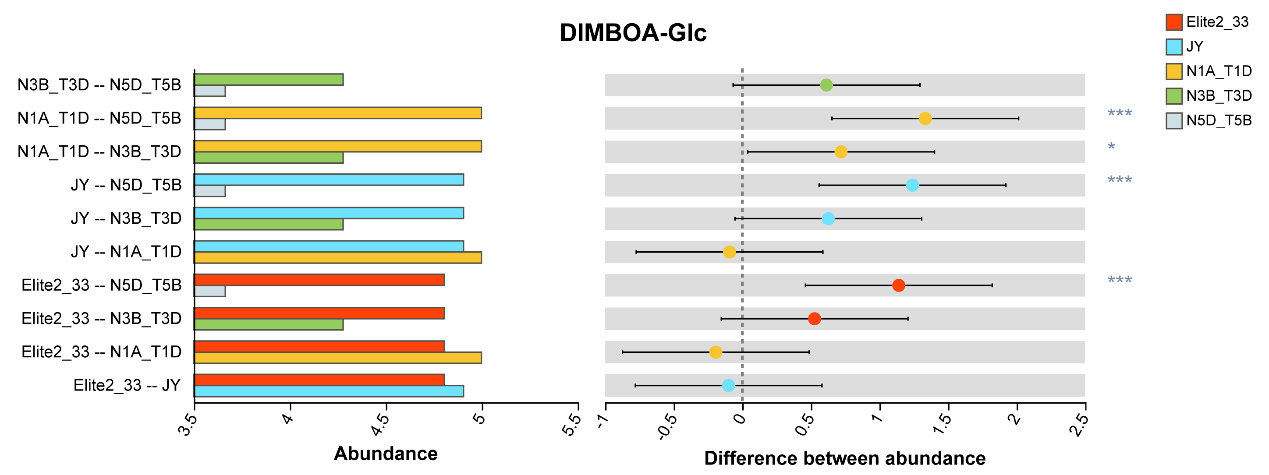


Figure S10 The average abundance of DIMBOA-Glu in different genotypes. * 0.01 < *P* ≤ 0.05, ** 0.001 < *P* ≤ 0.01, *** *P* ≤ 0.001.

Figure S11


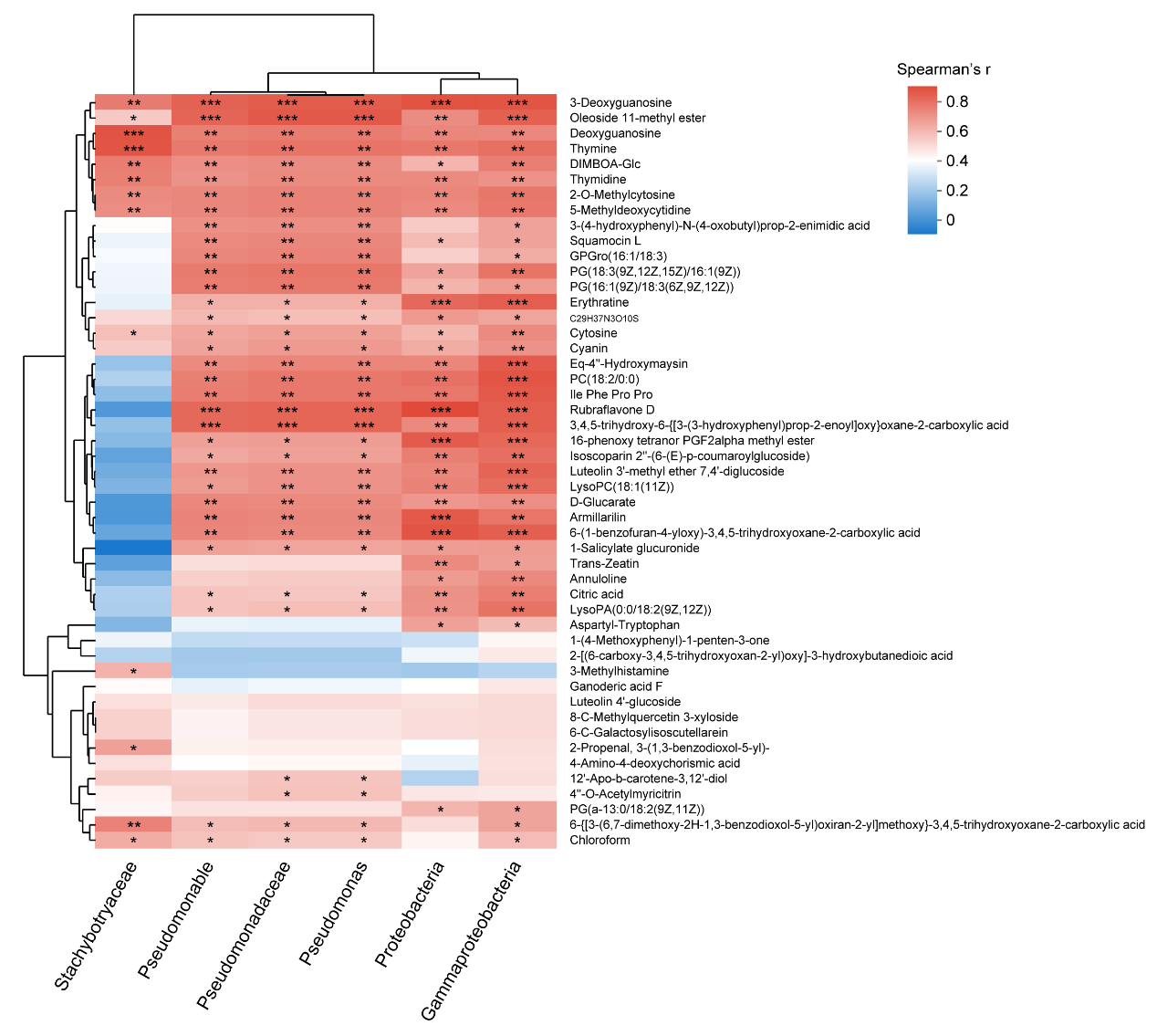


Figure S11 Heatmap shows the correlation between leaf metabolites and enriched bacterial taxa in phyllosphere.

Figure S12


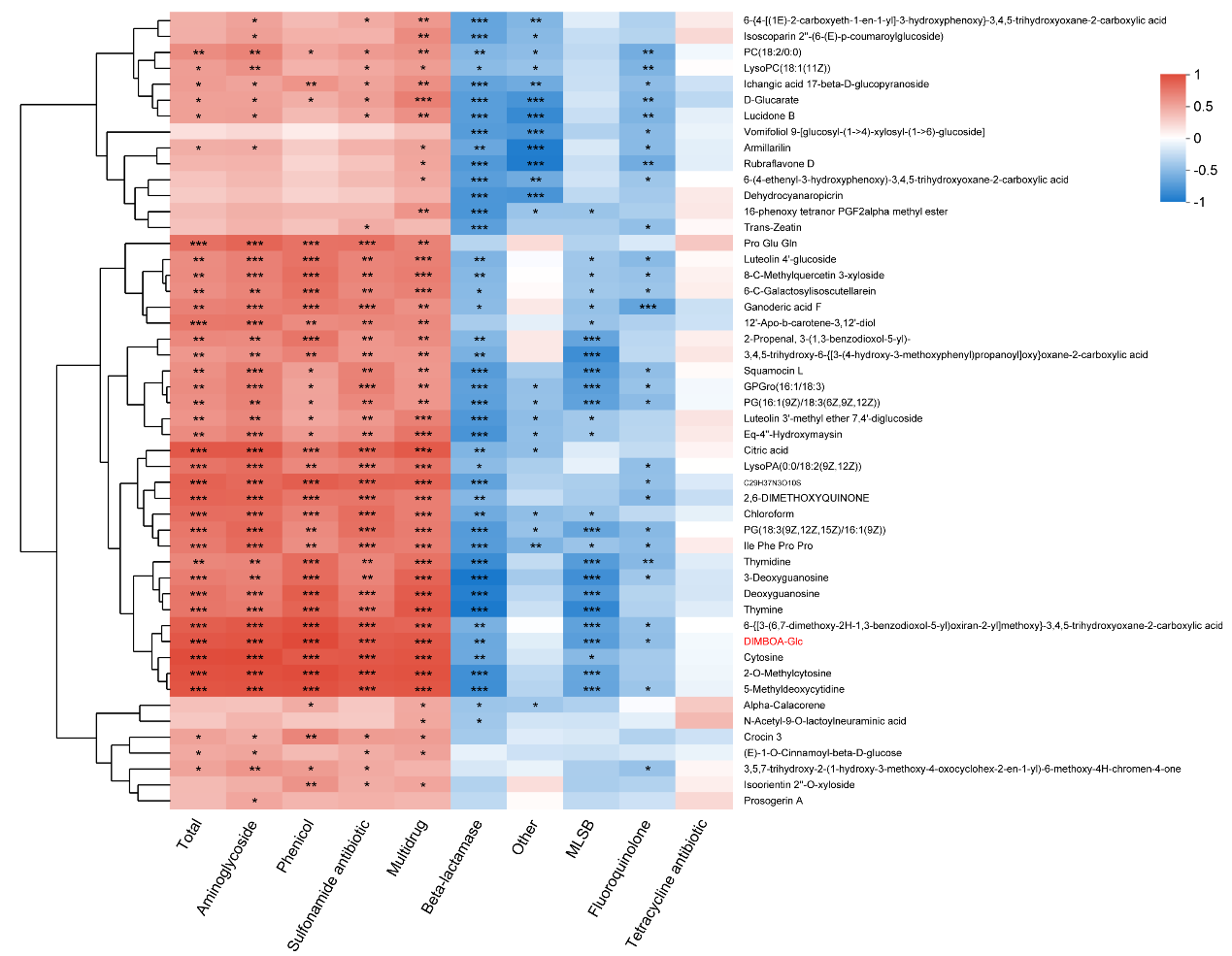


Figure S12 Heatmap shows the correlation between leaf metabolites and different classes ARGs in phyllosphere.

**Figure S13**


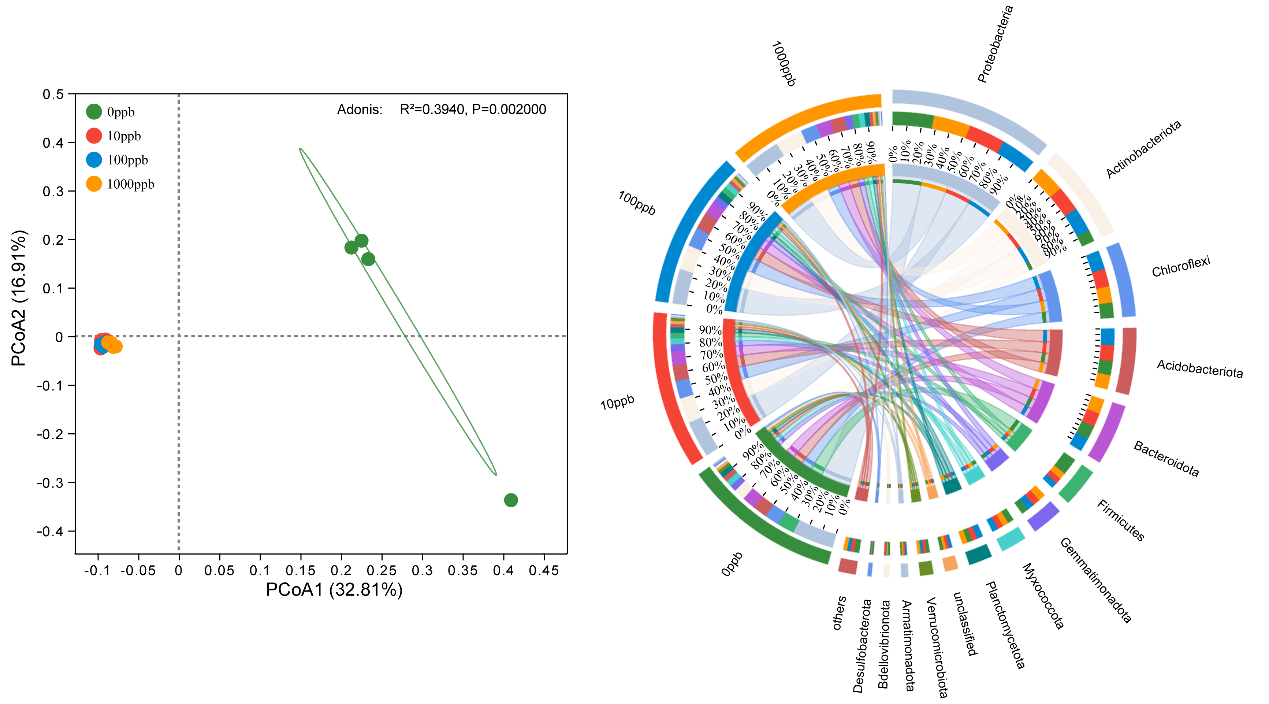


Figure S13 Principal coordinate analysis (PCoA) based on Bray−Curtis distances depicting the distinct distribution patterns of bacterial communities. Circos graph showing the microbial compositions in different DIMBOA-Glu concentrations.

**Figure S14**


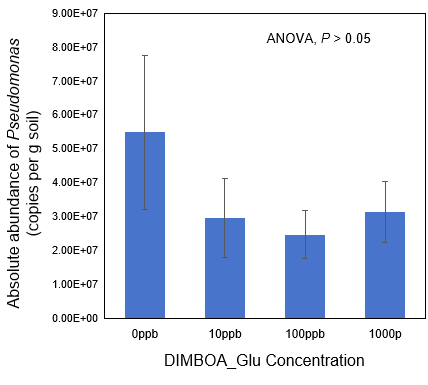


Figure S14 Microcosm experiment shows the influence of DIMBOA-Glu concentrations on the absolute abundance of *Pseudomonas* species.

**Table S1** The description of selected wheat cultivars

| **Number** | **Name** | **Description** |
| --- | --- | --- |
| 1 | CS | Chinese spring wheat; the standard cultivar for wheat cytogenetic research[21] |
| 2 | JY | Wheat cultivar in Jiyuan, Henan province, northern China |
| 3 | SC42 | Wheat cultivar in Sichuan province, southern China |
| 4 | Elite2-33 | Elite-II synthetic hexaploid wheat |
| 5 | N1D-T1B | Chromosome 1D is missing; chromosome 1B is 4; and 2 normal 1A chromosome |
| 6 | N1A-T1B | Chromosome 1A is missing; chromosome 1B is 4; and 2 normal 1D chromosome |
| 7 | N1A-T1D | Chromosome 1A is missing; chromosome 1D is 4; and 2 normal 1B chromosome |
| 8 | N2D-T2B | Chromosome 2D is missing; chromosome 2B is 4; and 2 normal 2A chromosome |
| 9 | N3B-T3D | Chromosome 3B is missing; chromosome 3D is 4; and 2 normal 3A chromosome |
| 10 | N3D-T3B | Chromosome 3D is missing; chromosome 3B is 4; and 2 normal 3A chromosome |
| 11 | N4A -T4B | Chromosome 4A is missing; chromosome 4B is 4; and 2 normal 4D chromosome |
| 12 | N4A -T4D | Chromosome 4A is missing; chromosome 4D is 4; and 2 normal 4B chromosome |
| 13 | N5B-T5A | Chromosome 5B is missing; chromosome 5A is 4; and 2 normal 5D chromosome |
| 14 | N5D-T5B | Chromosome 5D is missing; chromosome 5B is 4; and 2 normal 5A chromosome |
| 15 | N6D-T6B | Chromosome 6D is missing; chromosome 6B is 4; and 2 normal 6A chromosome |
| 16 | N7D-T7B | Chromosome 7D is missing; chromosome 7B is 4; and 2 normal 7A chromosome |

**Table S2** Information of 384 genes primers

| Gene_target | Forward primer | Reverse Primer | Drug Class |
| --- | --- | --- | --- |
| 16S | GGGTTGCGCTCGTTGC | ATGGYTGTCGTCAGCTCGTG | taxonomic |
| *Acinetobacter baumannii(ompA)* | TCTTGGTGGTCACTTGAAGC | ACTCTTGTGGTTGTGGAGCA | taxonomic |
| AAC(3)-Ia | ACGTTCTGCCAAAGTTTGAG | ACTGCCGGATCGTCAC | aminoglycoside antibiotic |
| AAC(3)-Ib | CAGCGAGACGTTCATCGC | CACGCTTCAGGTGGCTAATC | aminoglycoside antibiotic |
| AAC(3)-Id | AGATAGTTATGCCCGCAACAAG | ACGCGCTGCGCCTATA | aminoglycoside antibiotic |
| AAC(3)-Iic | ACGGCATTCTCGATTGCTTT | CCGAGCTTCACGTAAGCATTT | aminoglycoside antibiotic |
| AAC(3)-IId_IIa_Iie | CGATGGTCGCGGTTGGTC | TCGGCGTAGTGCAATGCG | aminoglycoside antibiotic |
| AAC(3)-IV | CCAACACGACGCTGCATC | GCTGTCGCCACAATGTCG | aminoglycoside antibiotic |
| AAC(3)-Via | GTGTCCGTCGCCAAGGA | GGTGACGGCCTTGTCGA | aminoglycoside antibiotic |
| AAC(3)-Xa | GCAAGCGGTTCGTGACGTA | TCAGGTGCTCCTCGATCCAG | aminoglycoside antibiotic |
| AAC(6')-Ib | CGTCGCCGAGCAACTTG | CGGTACCTTGCCTCTCAAACC | aminoglycoside antibiotic |
| AAC(6')-Ie-APH(2'')-Ia | CCAAGAGCAATAAGGGCATACCAA | GCCACACTATCATAACCACTACCG | aminoglycoside antibiotic |
| AAC(6')-Ig | GCGATGTTAGAAGCCTCAATTCG | CACACTTCGGCCTGTCGAA | aminoglycoside antibiotic |
| AAC(6')-IIa | CGACCCGACTCCGAACAA | GCACGAATCCTGCCTTCTCA | aminoglycoside antibiotic |
| AAC(6')-IIc | CAGTCTTTGGCTAATCCATCACAG | AACGAACCCGGCCTTCTC | aminoglycoside antibiotic |
| AAC(6')-Ij | ATGCCTGTATCTGAATCCCTGATG | GGCAATCGCTTGTTGAGTATCTG | aminoglycoside antibiotic |
| AAC(6')-Im | CGTGAGCATTATACAGAGCAATGG | CCATTTCCGTTCGTAGATATTGGC | aminoglycoside antibiotic |
| AAC(6')-Ip | GGGAATTATCGGAATAGCTCTTGG | TTGGGCTGTTCTTCCTAGCTAA | aminoglycoside antibiotic |
| AAC(6')-Ir | GCTATAACGATCAGCAGCAAGC | CGCGATGCATGGCATGAC | aminoglycoside antibiotic |
| AAC(6')-Is | AAGCTTACTCTGGCCTGATCATG | TGCCTGAACGTCGATATTCAGG | aminoglycoside antibiotic |
| AAC(6')-Iv | TTGGCTTATACCGACACCCA | CCCGTTGCGATACCTGAAC | aminoglycoside antibiotic |
| AAC(6')-Iw | TGCGTCAGTTACTTACACGAAC | CCTGATGCATTGCATGACTGA | aminoglycoside antibiotic |
| AAC(6')-Iy | GCCTCAATCCGCCACGATTA | ACGCGCTCTGTTTCCTCAAA | aminoglycoside antibiotic |
| AAC(6')-Iz | TGCGCCATGACTACGTGAAC | GACTGTCCGAAGCCAGTTCG | aminoglycoside antibiotic |
| aacA_aphD | AGAGCCTTGGGAAGATGAAGTTT | TTGATCCATACCATAGACTATCTCATCA | aminoglycoside antibiotic |
| aacA43 | CTTGGCCTACATTAGATTCAGCTC | GCTCTCAATCTTTGATAGGAGCAG | aminoglycoside antibiotic |
| aadA | GTTGTGCACGACGACATCATT | GGCTCGAAGATACCTGCAAGAA | aminoglycoside antibiotic |
| aadA10 | ACAGGCACTCAACGTCATCG | CGCGGAGAACTCTGCTTTGA | aminoglycoside antibiotic |
| aadA16 | ACGGTGGCCTGAAGCC | GAATTGCAGTTCCCGTCTGG | aminoglycoside antibiotic |
| aadA17 | TGTACGGCTCCGCAGTG | CACGGAATGATGTCGTCGTG | aminoglycoside antibiotic |
| aadA2 | CAATGACATTCTTGCGGGTATC | GACCTACCAAGGCAACGCTATG | aminoglycoside antibiotic |
| aadA21 | ACGGCTCCGCAGTGGAT | GGCCACAGTAACCAACAAATCA | aminoglycoside antibiotic |
| aadA5 | ATCACGATCTTGCGATTTTGCT | CTGCGGATGGGCCTAGAAG | aminoglycoside antibiotic |
| aadA6 | CCATCGAGCGTCATCTGGAA | CCCGTCTGGCCGGATAAC | aminoglycoside antibiotic |
| aadA7 | CACTCCGCGCCTTGGA | TGTGGCGGGCTCGAAG | aminoglycoside antibiotic |
| aadA9 | CGCGGCAAGCCTATCTTG | CAAATCAGCGACCGCAGACT | aminoglycoside antibiotic |
| ACC-1 | CACACAGCTGATGGCTTATCTAAAA | AATAAACGCGATGGGTTCCA | beta-lactamase |
| acrA | GGTCTATCACCCTACGCGCTATC | GCGCGCACGAACATACC | multidrug |
| acrB | AGTCGGTGTTCGCCGTTAAC | CAAGGAAACGAACGCAATACC | multidrug |
| acrF | GCGGCCAGGCACAAAA | TACGCTCTTCCCACGGTTTC | multidrug |
| acrR | GCGCTGGAGACACGACAAC | GCCTTGCTGCGAGAACAAA | multidrug |
| ACT beta-lactamase | AAGCCGCTCAAGCTGGA | GCCATATCCTGCACGTTGG | beta-lactamase |
| ADC beta-lactamase | GGTATGGCTGTGGGTGTTATTCA | AGGCAAGGTTACCACTTGTATACG | beta-lactamase |
| adeA | CAGTTCGAGCGCCTATTTCTG | CGCCCTGACCGACCAAT | multidrug |
| adeI | CAGTCTGGTTTGCAGTAACCA | CACTCCTACAACAACAGGCAA | multidrug |
| AmpC beta-lactamase | CAGGATCTGATGTGGGAGAACTA | TCGGGAACCATTTGTTGGC | beta-lactamase |
| ANT(2'')-Ia | CCTGCTTGGTGGGCAGAC | CGGCACGCAAGACCTCAA | aminoglycoside antibiotic |
| ANT(4') | CCGACAACATTTCTACCATCCTT | ACCGAAGCGCTCGTCGTATA | aminoglycoside antibiotic |
| ANT(4')-Ia | GATGGCCGCTGACACATG | TCAACATTGCGCCATAGTGG | aminoglycoside antibiotic |
| ANT(6) | TACCTTATTGCCCTTGGAAGAGTTA | GGAACTATGTCCCTTTTAATTCTACAATCT | aminoglycoside antibiotic |
| ANT(6)-Ia | TCGCCATGAGCTGCTGA | CCTATCATACTCCGGATAGGCATA | aminoglycoside antibiotic |
| ANT(6)-Ib | AGAACATCCGACAGCACGTTC | CCAACCTTCCATGAAATCATTCGC | aminoglycoside antibiotic |
| APH(2'')-Iva | TGAGCAGTATCATAAGTTGAGTGAAAAG | GACAGAACAATCAATCTCTATGGAATG | aminoglycoside antibiotic |
| APH(3')-Ia | TGAACAAGTCTGGAAAGAAATGCA | CCTATTAATTTCCCCTCGTCAAAAA | aminoglycoside antibiotic |
| APH(3'')-Ia | TAACAGCGATCGCGTATTTCG | TCCGACTCGTCCAACATCAATA | aminoglycoside antibiotic |
| APH(3')-Ib | AACAGGTTTGGGAGGCGATG | CGCAACAAGCCTCTCCTGAA | aminoglycoside antibiotic |
| APH(3')-Via | TCTCATGGCGATATCACGGATAG | TTTCCTCCGATGCATCCTCTC | aminoglycoside antibiotic |
| APH(3')-VIIa | CTCTCTCATGGAGATATGAGCGCTA | AATCCGGTTCAAGTCCCAACATG | aminoglycoside antibiotic |
| APH(3')-VIIIa | TCGGTATCCCGGTTGTGAG | ACACGAGGTACGGGAATCC | aminoglycoside antibiotic |
| APH(4)-Ia | CGCTCCCGATTCCGGAA | CACAGTTTGCCAGTGATACACA | aminoglycoside antibiotic |
| APH(4)-Ib | GGGAACACCGTGCTCACC | GTTGGTCCCGTGCAGGTC | aminoglycoside antibiotic |
| APH(6)-Ia | CGCTGGGAGCTGAAGAGG | AGCATCGTGCTGCTCTCC | aminoglycoside antibiotic |
| APH(6)-Ic | CACGACAACGTGCTCGAC | CCGTCTTCGGCGAACCA | aminoglycoside antibiotic |
| APH(6)-Id | GCTCGGTCGTGAGAACAATCT | CAATTTCGGTCGCCTGGTAGT | aminoglycoside antibiotic |
| APH(9)-Ib | GCTATGTGCTGGTGGACTGG | GGAACCACTCGACGAACTCG | aminoglycoside antibiotic |
| APH3-III | CAGAAGGCAATGTCATACCACTTG | GACAGCCGCTTAGCCGAA | aminoglycoside antibiotic |
| APHA3 | AAAAGCCCGAAGAGGAACTTG | CATCTTTCACAAAGATGTTGCTGTCT | aminoglycoside antibiotic |
| apmA | GGCGCACATGCATTCATCA | CTATACTCCAGTCCCACCATTTGA | aminoglycoside antibiotic |
| armA | TCTTCGACGAATGAAAGAGTCG | GCTAATGGATTGAAGCCACAACC | aminoglycoside antibiotic |
| arr-2 | TTGGCGATTGGTGACTTGCTAA | ATCGTCTTCGAACGGTCCTG | rifamycin antibiotic |
| arr-3 | GATCGTCTTCGAACGGTCCTG | TTTGGCGATTGGTGACTTGCT | rifamycin antibiotic |
| arsA | CAGGTCAGCCGCATCAACC | GCCTGAAACACGGCAATTTCTTC | multidrug |
| bacA | ATCCGCGGCACCCTGA | CCTGCTTGATGGACTTGATGAAGA | peptide antibiotic |
| Bacteroidetes | GGARCATGTGGTTTAATTCGATGAT | AGCTGACGACAACCATGCAG | taxonomic |
| BEL beta-lactamase | ATGTCCATGGCACAGACTGTG | CCTGTCTTGTCACCCGTTACC | beta-lactamase |
| bl1acc | TGTTATCCGTGATTACCTGTCTGG | CTCAGCGAGCCAACTTCAAATA | beta-lactamase |
| Bla1 | GCAAGTTGAAGCGAAAGAAAAGA | TACCAGTATCAATCGCATATACACCTAA | beta-lactamase |
| BlaB beta-lactamase | CGTGCCGGAGGTCTTGAATA | GGGATAGTAAACCTGAAACTCGGA | beta-lactamase |
| blaSFO | CCGCCGCCATCCAGTA | GGGCCGCCAAGATGCT | beta-lactamase |
| blaZ beta-lactamase | GGAGATAAAGTAACAAATCCAGTTAGATATGA | TGCTTAATTTTCCATTTGCGATAAG | beta-lactamase |
| cadC | CGCTCTGTGTCAGGATGAAGAG | CTTTCTTATGTGCTAGGGCGATCA | multidrug |
| CARB beta-lactamase | TGATTTGAGGGATACGACAACTCC | CTGTAATACTCCGAGCACCAA | beta-lactamase |
| CARB-2 | TTGTGACCTATTCCCCTGTAATAGAA | TGCGAAGCACGCATCATC | beta-lactamase |
| cat | ATCGGCCAGACTGGATATCGA | CACAGCTCCAGTTGCAACAAC | phenicol antibiotic |
| cat(pC221) | AATGACCGTATGCTGCAAGAAG | TTTGCCTGCTATGGCATTCTG | phenicol antibiotic |
| catB2 | GCTACTATTCCGGCTATTACCATG | GGGCTCCTCGTTCATGTAGA | phenicol antibiotic |
| catB3 | GCACTCGATGCCTTCCAAAA | AGAGCCGATCCAAACGTCAT | phenicol antibiotic |
| catB8 | CACTCGACGCCTTCCAAAG | CCGAGCCTATCCAGACATCATT | phenicol antibiotic |
| catB9 | CACCTTATGAAGTGGTCGGTTCA | GTCTGATGAACACAGAGACTGCA | phenicol antibiotic |
| catI | GGGTGAGTTTCACCAGTTTTGATT | CACCTTGTCGCCTTGCGTATA | phenicol antibiotic |
| catII | CCTGGAACCGCAGAGAACA | CGGAACTCCGGAAACTGATTAAC | phenicol antibiotic |
| catIII | CTGATTGCTCAGGCCGTGAA | ATGAGTATGGGCAACTCAGTGC | phenicol antibiotic |
| catP | CCTTTGGACTGAGTGTAAGTCTGA | TAAAGCCATCGAAGGTTGACCA | phenicol antibiotic |
| catQ | AGGTGCACTTACAGTATGACTGC | AACGTGGGAAGTTCTCGTCATAC | phenicol antibiotic |
| CcrA | GCAGCGTTGCTGGACACA | GTTCGGGATAAACGTGGTGACT | beta-lactamase |
| CcrA beta-lactamase | CACTGGCACGGCGATTGTA | CGGCAGCCAAACCACGATA | beta-lactamase |
| cefa_qacelta | TAGTTGGCGAAGTAATCGCAAC | TGCGATGCCATAACCGATTATG | multidrug |
| ceoA | ATCAACACGGACCAGGACAAG | GGAAAGTCCGCTCACGATGA | multidrug |
| cepA beta-lactamase | AGTTGCGCAGAACAGTCCTCTT | TCGTATCTTGCCCGTCGATAAT | beta-lactamase |
| Cfr Group | GCAAAATTCAGAGCAAGTTACGAA | AAAATGACTCCCAACCTGCTTTAT | Multidrug |
| CfxA beta-lactamase | TCATTCCTCGTTCAAGTTTTCAGA | TGCAGCACCAAGAGGAGATGT | beta-lactamase |
| class C beta-lactamase | CTGGCGCATACCTGGATTAC | GCCAGTTCAGCATCTCCCA | beta-lactamase |
| cmlA1 | TAGGAAGCATCGGAACGTTGAT | CAGACCGAGCACGACTGTTG | phenicol antibiotic |
| cmlA5 | GCGCTCTTCGAGGATTCG | CCGCCCAAGCAGAAGTAGAC | phenicol antibiotic |
| cmlv | GCCCTCATCACCGTCTTCG | GGACGTTGGCGATGGAGAG | phenicol antibiotic |
| cmr | CGGCATCGTCAGTGGAATT | CGGTTCCGAAAAAGATGGAA | multidrug |
| cmx | GCGATCGCCATCCTCTGT | TCGACACGGAGCCTTGGT | phenicol antibiotic |
| CMY beta-lactamase | AAAGCCTCATGGGTGCATAAA | ATAGCTTTTGTTTGCCAGCATCA | beta-lactamase |
| CMY_MOX beta-lactamase | CTATGTCAATGTGCCGAAGCA | GGCTTGTCCTCTTTCGAATAGC | beta-lactamase |
| copA | TGCACCTGACVGGSCAYAT | GVACTTCRCGGAACATRCC | multidrug |
| CphA beta-lactamase | GCGAGCTGCACAAGCTGAT | CGGCCCAGTCGCTCTTC | beta-lactamase |
| cphA2 | GTAACGCCTACTGGAAGTCCA | CAGCTTCTCCTTGAGAATGCAG | beta-lactamase |
| cro | AGATGTTATCGACCACTTCGGA | CCGCTTGGCGATAAGCG | MGE |
| CTX-M beta-lactamase | GCGATAACGTGGCGATGAAT | GTCGAGACGGAACGTTTCGT | beta-lactamase |
| CTX-M-1_3_15 | CGTACCGAGCCGACGTTAA | CAACCCAGGAAGCAGGCA | beta-lactamase |
| czcA | GCCTTGTTCATCGGCGAAC | GGCAATGTCGCCTTCGTTC | multidrug |
| dfrA1 | GGAATGGCCCTGATATTCCA | AGTCTTGCGTCCAACCAACAG | diaminopyrimidine antibiotic |
| dfrA10 | CTTCAACTATCACAGAGCACGAAG | TCTACCGGTACATACACATCAGC | diaminopyrimidine antibiotic |
| dfrA12 | CCTCTACCGAACCGTCACACA | GCGACAGCGTTGAAACAACTAC | diaminopyrimidine antibiotic |
| dfrA14 | CGGATCATGTCATTGTTTCAGG | ATGTTAGAGGCGAAGTCTTGG | diaminopyrimidine antibiotic |
| dfrA15 | AGGCCGAAAGACTTTCGAGTC | TCACCTTCTGGCTCAATGTCG | diaminopyrimidine antibiotic |
| dfrA17 | CGGGAACGGCCCTGATATTCC | CGTGTTGCGACCGCATACTTTC | diaminopyrimidine antibiotic |
| dfrA18 | GGAGCGAATCAAGGAGAAAGGAA | GCAATGCGTTGATCGGTATTCTC | diaminopyrimidine antibiotic |
| dfrA21 | TTGTTTCAACGCTGTCGCA | GGTTTCGGTTGAGACAAGCTC | diaminopyrimidine antibiotic |
| dfrA22 | CAGCCGAACACGGCAAAG | CGGAGTGCGTGTACGTGA | diaminopyrimidine antibiotic |
| dfrA25 | TCAAACTGGACAGCGGCTA | GTCGATTGTCGACACATGCA | diaminopyrimidine antibiotic |
| dfrA27 | GCCGCTCAGGATCGGTA | GTCGAGATATGTAGCGTGTCG | diaminopyrimidine antibiotic |
| dfrA5 | CCATGGAGTGCCAAAGGTG | CACCTTTGGCACTCCATGG | diaminopyrimidine antibiotic |
| dfrA7 | GTAATCGGTAGTGGTCCTGA | ATCAGGACCACTACCGATTAC | diaminopyrimidine antibiotic |
| dfrA8 | GGTCGCACCTGCATCGTTA | AGCGCCACCAATGACGTAG | diaminopyrimidine antibiotic |
| dfrB4 | CGGTTCGCATTCCCATCAAA | CGCAGTCATGGGATAAATCTGG | diaminopyrimidine antibiotic |
| dfrBmulti | ACCAAGGCAGAAGTGAAGTCA | GGTGAGCCTCAGACTCGAC | diaminopyrimidine antibiotic |
| dfrC | GTCGCTCACGATAAACAAAGAGTC | CCCTTCATGGTGAAATGAAGCTTG | diaminopyrimidine antibiotic |
| dfrG | TCAATCGGAAGAGCCTTACCTGA | TGGGCAAATACCTCATTCCATTCC | diaminopyrimidine antibiotic |
| dfrK | TGCTGCGATGGATAAGAACAG | CTTCCAGGTAATGCTCTTCCG | diaminopyrimidine antibiotic |
| DHA beta-lactamase | TGGCCGCAGCAGAAAGA | CCGTTTTATGCACCCAGGAA | beta-lactamase |
| EAE_05855 | CCCATCACCGCTGAACTGG | TGGGCGCTGCCATCTAAAC | MGE |
| emrB_qacA | CTTTTCTCTAACCGTACATTATCTACGATAAA | AGAACGTAGCGACTGATAAAATGCT | multidrug |
| emrD | CTCAGCAGTATGGTGGTAAGCATT | ACCAGGCGCCGAAGAAC | multidrug |
| Enterococci(23S) | AGAAATTCCAAACGAACTTG | CAGTGCTCTACCTCCATCATT | taxonomic |
| EreA | GATAATTCTGCTGGCGCACA | GCAGGCGTGGTCACAAC | MLSB |
| EreB | TCGTATATGGCGGGCGTAGTA | GGTCCAAGATGGGTGAATGCA | MLSB |
| Erm(34) | AAAGCGGTTTACAAGCGTTTCG | GGGTGCTCTAGGGTTGTTTAGTG | MLSB |
| Erm(35) | CCTTCAGTCAGAACCGGCAA | GCTGATTTGACAGTTGGTGGTG | MLSB |
| Erm(36) | GGCGGACCGACTTGCAT | TCTGCGTTGACGACGGTTAC | MLSB |
| Erm(42) | TGTTGAGATTGGGCCTGGA | CTAAGGGTGGGTTCTCACTATCTA | MLSB |
| Erm(K) | GTTTGATATTGGCATTGTCAGAGAAA | ACCATTGCCGAGTCCACTTT | MLSB |
| erm(O) | TGATGACGGCTCAGTGG | GTGCACCAGCGCCTGA | MLSB |
| ErmA | TCGTTGAGAAGGGATTTGCGA | TTGCATGCTTCAAAGCCTGTC | MLSB |
| ermA_ermTR | ACATTTTACCAAGGAACTTGTGGAA | GTGGCATGACATAAACCTTCATCA | MLSB |
| ErmB | GAACACTAGGGTTGTTCTTGCA | CTGGAACATCTGTGGTATGGC | MLSB |
| ErmD | TTTCCGGACAGCATTTGATGC | TCCACTGCCAATACCTTACCG | MLSB |
| ErmE | GTCACGCAGCTGGAGTTCG | CGGTGAAGCACAGCTCGAC | MLSB |
| ErmF | CAGCTTTGGTTGAACATTTACGAA | AAATTCCTAAAATCACAACCGACAA | MLSB |
| ErmG | CCCTTGAATTAGTACAGAGGTG | GCAAACTCGTATTCCACGA | MLSB |
| ErmH | GGAGTGAGGCTGACCGTAGAAG | ATCGGCGAAACGCACAAA | MLSB |
| ErmQ | TGAAAGCCATGCGTCTGAC | TTCAGCTGGCAGCTTAAGC | MLSB |
| ErmS | GAGTACGCCCGCAAACG | GCGTTCGATCCGGAGGA | MLSB |
| ErmT | GTTCACTAGCACTATTTTTAATGACAGAAGT | GAAGGGTGTCTTTTTAATACAATTAACGA | MLSB |
| ErmX | GCTCAGTGGTCCCCATGGT | ATCCCCCCGTCAACGTTT | MLSB |
| ErmY | TTGTCTTTGAAAGTGAAGCAACAGT | TAACGCTAGAGAACGATTTGTATTGAG | MLSB |
| fabK | CAGGAGCAGGAAATCCAAGC | CCAGCTTCCATTCCTTCTGC | other |
| fexA | TGGTGTGGCTGTTGCAATCTTA | CCAAGGTACAAAGCACCTTGGA | phenicol antibiotic |
| Firmicutes | GGAGYATGTGGTTTAATTCGAAGCA | AGCTGACGACAACCATGCAC | taxonomic |
| floR | AACCCGCCCTCTGGATCA | GCCGTCGAGAAGAAGACGAA | phenicol antibiotic |
| folA | CGAGCAGTTCCTGCCAAAG | CCCAGTCATCCGGTTCATAATC | other |
| FosB | CTTGCAGGCCTATGGATTGC | TCTGTTCTCAAGTGTGCCAGTA | fosfomycin |
| FosX | AGCTGGTTTGTGGATTTGCA | CCACACCGAGAGCTTTAATCCG | fosfomycin |
| FOX beta-lactamase | CCTACGGCTATTCGAAGGAAGATAAG | CCGGATTGGCCTGGAAGC | beta-lactamase |
| GES beta-lactamase | GCAATGTGCTCAACGTTCAAG | GTGCCTGAGTCAATTCTTTCAAAG | beta-lactamase |
| GOB beta-lactamase | CTTGGGCTTGAATGCTCAGGTA | TGTATGGTCGTAGTGAGCCTGA | beta-lactamase |
| HERA beta-lactamase | GGGCAACCGCATTCTGAC | GCATCTCCCACTTTATCGTCAC | beta-lactamase |
| IMI beta-lactamase | ACATCTACACCTGCAGCAGTAG | AATCGCTTGGTACGCTAGCA | beta-lactamase |
| IMIR beta-lactamase | AGCCGGACTAGAGCTTCATG | GGCAGAACTCATCATCTGCAAA | beta-lactamase |
| IMP beta-lactamase | GGAATAGAGTGGCTTAATTC | GGTTTAACAAAACAACCACC | beta-lactamase |
| IncHI2-smr0018 | ATAATGATTCACCGGGGTAG | CTTCAGGCTATCGTTTCG | MGE |
| IncI1_repI1 | CGAAAGCCGGACGGCAGAA | TCGTCGTTCCGCCAAGTTCGT | MGE |
| IncN_korA | GGAACGTTTGTAYCTTGTATTG | ACTCACTATCTTCTGTTGATTG | MGE |
| IncN_oriT | TTGGGCTTCATAGTACCC | GTGTGATAGCGTGATTTATGC | Plasmid-inc |
| IncN_rep | AGTTCACCACCTACTCGCTCCG | CAAGTTCTTCTGTTGGGATTCCG | Plasmid-inc |
| IncP_oriT | CAGCCTCGCAGAGCAGGAT | CAGCCGGGCAGGATAGGTGAAGT | Plasmid-inc |
| IncQ_oriT | TTCGCGCTCGTTGTTCTTCGAGC | GCCGTTAGGCCAGTTTCTCG | Plasmid-inc |
| IncW_trwAB | AGCGTATGAAGCCCGTGAAGGG | AAAGATAAGCGGCAGGACAATAACG | Plasmid-inc |
| IND beta-lactamase | CGCCTGTTAAACCCAACCTGTA | CGCTCTGTCATCATGAGAGTGG | beta-lactamase |
| intI1_337old | GCCTTGATGTTACCCGAGAG | GATCGGTCGAATGCGTGT | Integrase |
| intl2 | TGCTTTTCCCACCCTTACC | GACGGCTACCCTCTGTTATCTC | Integrase |
| intl3_339old | GCCACCACTTGTTTGAGGA | GGATGTCTGTGCCTGCTTG | Integrase |
| IS1111 | GTCTTAAGGTGGGCTGCGTG | CCCCGAATCTCATTGATCAGC | Insertional |
| IS1133 | GCAGCGTCGGGTTGGA | ACGCGTTCGAACAACTGTAATG | Insertional |
| IS1247 | CGGCCGTCACTGACCAA | TCGGCAGGTTGGTGACG | MGE |
| IS15DI | CAATACCTTTGATGGTGGCGTAAG | CTTACGCCACCATCAAAGGTATTG | MGE |
| IS200 | CCAAATACCGAAGACAAGCGTTC | CCAAACTGCTCGTAAAGCATCAG | MGE |
| IS200 | GCACACCCGATGGAACTGTAAA | TCGGCGGGATCTCCAGAAG | MGE |
| IS21-ISAs29 | GGTCCGTCAGGCACAAGTC | GGGATCGTATCGGCAAGCC | MGE |
| IS256 | CTTGCGCATCATTGGATGATGG | AAGAACGGCTCCAATTAAGCGA | MGE |
| IS26 | ATGGATGAAACCTACGTGAAGGTC | CGGTACTTAATCTGTCGGTGTTCA | MGE |
| IS3 | CGGTCTGAGCTTCGGGAA | AGAACTGTCACTCCGGTCTG | MGE |
| IS5/IS1182 | TTCTCGAAGAATCGCCATGGC | GCTTTGGATCGCTCCAATCGA | MGE |
| IS6/257 | ATATCGTGCCATTGATGCAGAG | ACCATTGCTACCTTCGTTGAAG | MGE |
| IS6100 | CGCACCGGCTTGATCAGTA | CTGCCACGCTCAATACCGA | MGE |
| IS613 | AGGTTCGGACTCAATGCAACA | TTCAGCACATACCGCCTTGAT | Transposase |
| IS630 | CCGCCACCAGTGTGATGG | TTGGCGCTGACTGGATGC | MGE |
| IS91 | GGATGCCACTGCTGGTCA | ACAGTGGATACAGTATCTGCTGAG | MGE |
| ISAba3-Acineto | TCAGAGGCAGCGGTATACGA | GGTTGATTCAGTTAAAGTACGTAAAACTTT | Insertional |
| ISCR1 | ATGGTTTCATGCGGGTT | CTGAGGGTGTGAGCGAG | MGE |
| ISEcp1 | CATGCTCTGCGGTCACTTC | GACGCACCTTCTTGATGACC | MGE |
| ISEfm1-Entero | AGGTGTCCATGACGTGAAAGTG | TCCTTTGTCCCCTAGGATATTGG | Insertional |
| ISPps1-pseud | CACACTGCAAAAACGCATCCT | TGTCTTTGGCGTCACAGTTCTC | Insertional |
| ISSm2-Xanthob | TGGATCGACCGGTTCCAT | GCTGACCGAGCTGTCCATGT | Insertional |
| *Klebsiella pneumoniae(gltA)* | ACGGCCGAATATGACGAATTC | AGAGTGATCTGCTCATGAA | taxonomic |
| KPC beta-lactamase | GCCGCCAATTTGTTGCTGAA | GCCGGTCGTGTTTCCCTTT | beta-lactamase |
| L1 beta-lactamase | CACCGGGTTACCAGCTGAAG | GCGAAGCTGCGCTTGTAGTC | beta-lactamase |
| LEN beta-lactamase | TGTTCGCCTGTGTGTTATCTCC | GCAGCACTTTAAAGGTGCTCAC | beta-lactamase |
| lmrA | TTCAGATGCAATGGCGTTTG | ATAATCGGGAACATAATGAGCATAACTAC | MLSB |
| lncF_FIC | GTGAACTGGCAGATGAGGAAGG | TTCTCCTCGTCGCCAAACTAGAT | MGE |
| lnuA | TGACGCTCAACACACTCAAAAA | TTCATGCTTAAGTTCCATACGTGAA | MLSB |
| lnuB | GGATCGTTTACCAAAGGAGAAGG | AGCATAGCCTTCGTATCAGGAA | MLSB |
| lnuC | GGGTGTAGATGCTCTTCTTGGA | CTTTACCCGAAAGAGTTTCTACCG | MLSB |
| lnuF | ATACCGGTCATTTCCACTTGGC | GCATCAGGCTGATGAGGTTCAA | MLSB |
| lsaC | AAACGGCGTGAAAGTATCAGG | TTGTGGTGATGTAACGGATGC | Multidrug |
| marR | GCTGTTGATGACATTGCTCACA | CGGCGTACTGGTGAAGCTAAC | multidrug |
| MCR-1.1 | CACATCGACGGCGTATTCTG | CAACGAGCATACCGACATCG | peptide antibiotic |
| MCR-2.1 | CGGCGTACTTTAAGCGTTATGATG | GCATTTGGCATACCATGCAGATAG | peptide antibiotic |
| mdtA | ACAAGCCCAGGGCCAAC | CCTTAATGGTGCCTTCGGTTTC | multidrug |
| mdtE | CGTCGGCGCACTCGTT | TCCAGACGTTGTACGGTAACCA | multidrug |
| mdtG | TTCCAGCCGGTCAGCAA | GACATCTCCCGCGAGTTCG | multidrug |
| mdtH | ATGCTGGCTGTACAAGTGATG | CACTCCAGCGGGCGATA | fluoroquinolone antibiotic |
| MdtK | TCGGGCATCCCGTTTATGATC | GTAGGCTGCGCATAATACCCA | multidrug |
| mecA | GGTTACGGACAAGGTGAAATACTGAT | TGTCTTTTAATAAGTGAGGTGCGTTAATA | beta-lactamase |
| mecA-Staphylococci | CGCAACGTTCAATTTAATTTTGTTAA | TGGTCTTTCTGCATTCCTGGA | taxonomic |
| mef(B) | CCGATAGGCTTACTTGTTGCAG | AGTCCACTTGCGGTTTCATTG | MLSB |
| mel | TAATTATCGCAGCAGCTGGTTC | GTTCCCAAACGGAGTATAAGAGTG | Multidrug |
| mel | GGCAAGCTAGGTGTTGAGC | ATTGCTCAACACCTAGCTTGC | Multidrug |
| mepA | ATCGGTCGCTCTTCGTTCAC | ATAAATAGGATCGAGCTGCTGGAT | multidrug |
| merA-marko | GTGCCGTCCAAGATCATG | GGTGGAAGTCCAGTAGGGTGA | multidrug |
| MexA | AGGACAACGCTATGCAACGAA | CCGGAAAGGGCCGAAAT | multidrug |
| MexB | CTGGAGATCGACGACGAGAAG | GAAATCGTTGACGTAGCTGGAA | multidrug |
| MexE | GGTCAGCACCGACAAGGTCTAC | AGCTCGACGTACTTGAGGAACAC | multidrug |
| MIR beta-lactamase | CGGTCTGCCGTTACAGGTG | AAAGACCCGCGTCGTCATG | beta-lactamase |
| mobA | GCTTCCCGTAACGAGGTAGT | CCTTGAACGGTATCAGCACG | MGE |
| mphA | TCAGCGGGATGATCGACTG | GAGGGCGTAGAGGGCGTA | MLSB |
| mphB | CGCAGCGCTTGATCTTGTAG | TTACTGCATCCATACGCTGCTT | MLSB |
| msrA | CTGCTAACACAAGTACGATTCCAAAT | TCAAGTAAAGTTGTCTTACCTACACCATT | Multidrug |
| msrC | TCAGACCGGATCGGTTGTC | CCTATTTTTTGGAGTCTTCTCTCTAATGTT | Multidrug |
| msrE | CGGCAGATGGTCTGAGCTTAAA | CGCACTCTTCCTGCATAAAGGA | Multidrug |
| mtrD | CGGAGTCCATCGACCATTTG | ATCGTCGGCAAGGAGAATCA | multidrug |
| mtrE | CGATGTGTCGTTTTGGAAGGT | CCTGCACCATGATTCCTCAATA | multidrug |
| multidrug resistance | AATTTTGCCGATTATTGCTGAAA | GATTGTCATCATTCGTTTATCACCAA | multidrug |
| NDM beta-lactamase | GGCCACACCAGTGACAATATCA | CAGGCAGCCACCAAAAGC | beta-lactamase |
| nimE | TGCGCCAAGATAGGGCATA | GTCGTGAATTCGGCAGGTTTA | multidrug |
| nisB | GGGAGAGTTGCCGATGTTGTA | AGCCACTCGTTAAAGGGCAAT | other |
| norA | ATCGCCGTTTGGTGGTACG | TCCACCAATCCCTGGTCCTAAA | multidrug |
| OCH beta-lactamase | GGCGACTTGCGCCGTAT | TTTTCTGCTCGGCCATGAG | beta-lactamase |
| oleC | CCCGGAGTCGATGTTCGA | GCCGAAGACGTACACGAACAG | MLSB |
| oprD | ATGAAGTGGAGCGCCATTG | GGCCACGGCGAACTGA | multidrug |
| optrA | GGTGGATGAAGTCCGTACGG | AGGTTAGACCTCCAAGAGCCA | Multidrug |
| oqxA | GAGTCAACCTACCTCCACTATCA | GCTGCGAGTTATCCAGCAG | multidrug |
| orf37-IS26 | GCCGGGTTGTGCAAATAGAC | TGGCAATCTGTCGCTGCTG | Insertional |
| orf39-IS26 | GCGCGTCGAGCATCAATAG | CAGTTGTGCTGCTGGTGGTC | Insertional |
| OXA-10 | CGACCGAGTATGTACCTGCTTC | TCAAGTCCAATACGACGAGCTA | beta-lactamase |
| OXY-1-1 | AAAGGTGACCGCATTCGC | CCAGCGTCAGCTTGCG | beta-lactamase |
| OXY-2-1 | CGTTCAGGCGGCAGGTT | GCCGCGATATAAGATTTGAGAATT | beta-lactamase |
| *Pseudomonas aeruginosa(ecfX)* | AGCGTTCGTCCTGCACAAGT | TCCACCATGCTCAGGGAGAT | taxonomic |
| pAKD1-IncP-1β | GGTAAGATTACCGATAAACT | GTTCGTGAAGAAGATGTA | Plasmid-rep |
| PAMBL-1-F_377old | CAGGCTCTTAATGTGATA | TTATGCTCAATACTCGTG | Plasmid-rep |
| pbp | CCGGTGCCATTGGTTTAGA | AAAATAGCCGCCCCAAGATT | beta-lactamase |
| Pbp5 | GGCGAACTTCTAATTAATCCTATCCA | CGCCGATGACATTCTTCTTATCTT | beta-lactamase |
| pbrT | GATGCGCACTGGGCTTG | TCGGAATATGCGGAAATGCG | multidrug |
| pBS228-IncP-1α | CAATCCATCGACAATCAC | GACAATCAGCTACTTCAC | Plasmid-rep |
| pcoA | TGGCGTATGGAGTTTCAATGC | GAATAATGCCGTGCCAGTGAA | multidrug |
| PDC beta-lactamase | CGCCGTACAACCGGTGAT | GAAGTAATGCGGTTCTCCTTTCA | beta-lactamase |
| penA | AGACGGTAACGTATAACTTTTTGAAAGA | GCGTGTAGCCGGCAATG | beta-lactamase |
| PER-1 | GCAAATGAAGCGCAGATGC | GACCACAGTACCAGCTGGTA | beta-lactamase |
| pica | GCAATCGAGGCGGTGTTC | TTGCCGCAGCCAATTCA | MLSB |
| pikR2 | TCGTGGGCCAGGTGAAGA | TTCCCCTTGCCGGTGAA | MLSB |
| pmrA | TTTGCAGGTTTTGTTCCTAATGC | GCAGAGCCTGATTTCTCCTTTG | fluoroquinolone antibiotic |
| qacA_B | AAGGGCCACTGCATTAGCTG | CCAGTCCAATCATGCCTGCA | fluoroquinolone antibiotic |
| qacF_H | TCGCAACATCCGCATTAAAA | ATGGATTTCAGAACCAGAGAAAGAAA | multidrug |
| qacH_351 | GTCGGTGTTGCTTATGCAGTCT | CAACCAGGCAATGGCTGTAA | multidrug |
| QepA_1_2 | GGGCATCGCGCTGTTC | GCGCATCGGTGAAGCC | fluoroquinolone antibiotic |
| QnrA | AGGATTTCTCACGCCAGGATT | CCGCTTTCAATGAAACTGCAA | fluoroquinolone antibiotic |
| QnrB4 | TCACCACCCGCACCTG | GGATATCTAAATCGCCCAGTTCC | fluoroquinolone antibiotic |
| QnrB46_47_48 | CGACGTTCAGTGGTTCAGATCTC | GCCAAGCCGCTCCATGAG | fluoroquinolone antibiotic |
| qnrB | GCGACGTTCAGTGGTTCAGA | GCTGCTCGCCAGTCGAA | fluoroquinolone antibiotic |
| QnrD | CGCTGGAATGGCACTGTGA | GCTCTCCATCCAACTTCACTCC | fluoroquinolone antibiotic |
| QnrS1_S3_S5 | CCACTTTGATGTCGCAGATCTTC | CCCTCTCCATATTGGCATAGGAAA | fluoroquinolone antibiotic |
| QnrS2 | TCCCGAGCAAACTTTGCCAA | GGTGAGTCCCTATCCAGCGA | fluoroquinolone antibiotic |
| QnrVC1_VC3_VC6 | CTCACATCAGGACTTGCAAGAA | ATGAAGCATCTCGAAGATCAGC | fluoroquinolone antibiotic |
| QnrVC4_VC5_VC7 | TTCCTTTAAACGGGCAAACCTC | CGATACCTGATTCATGAAGCTAGC | fluoroquinolone antibiotic |
| ROB-1 | GCAAAGGCATGACGATTGC | CGCGCTGTTGTCGCTAAA | beta-lactamase |
| SAT-4 | GAATGGGCAAAGCATAAAAACTTG | CCGATTTTGAAACCACAATTATGATA | nucleoside antibiotic |
| SHV-11 | TTGACCGCTGGGAAACGG | TCCGGTCTTATCGGCGATAAAC | beta-lactamase |
| silE | GGTGGAAAGTCATCAGAGGATGA | CAAAGCCCAGCAAGGATGC | multidrug |
| SME beta-lactamase | GAGGAAGACTTTGATGGGAGGATTG | CGCTATATTGCAATGCAGCAGAAG | beta-lactamase |
| spec_aph | GGTGCTGATATGAATGCCTTTGG | CATTGGGCGCATCAATAAATGG | aminoglycoside antibiotic |
| str | AATGAGTTTTGGAGTGTCTCAACGTA | AATCAAAACCCCTATTAAAGCCAAT | aminoglycoside antibiotic |
| strA | CCGGTGGCATTTGAGAAAAA | GTGGCTCAACCTGCGAAAAG | aminoglycoside antibiotic |
| sugE | CTTAGTTATTGCTGGTCTGCTGGA | GCATCGGGTTAGCGGACTC | multidrug |
| sul1 | GCCGATGAGATCAGACGTATTG | CGCATAGCGCTGGGTTTC | sulfonamide antibiotic |
| sul2 | TCATCTGCCAAACTCGTCGTTA | GTCAAAGAACGCCGCAATGT | sulfonamide antibiotic |
| sul3 | CGCGCTCAAGGCAGATG | GGGAATGCCATCTGCCTTG | sulfonamide antibiotic |
| sulA_folP | CAGGCTCGTAAATTGATAGCAGAAG | CTTTCCTTGCGAATCGCTTT | sulfonamide antibiotic |
| tcrB | GTGCCGGAACTCAAGTAGCA | GCACCGACTGCTGGACTTAA | multidrug |
| TEM beta-lactamase | CGCCGCATACACTATTCTCAG | GCTTCATTCAGCTCCGGTTC | beta-lactamase |
| terW | TCAAAGAGCTACGCGAGTCATA | CCTTCCCTGTGGACTCACC | multidrug |
| tet(38) | AAGCGACATTAGCCGGTTTAG | CTGCTCGTACTTAAGCCAAGG | tetracycline antibiotic |
| tet(39) | TATAGCGGGTCCGGTAATAGGTG | CCATAACGATCCTGCCCATAGATAAC | tetracycline antibiotic |
| tet(40) | CTGTCCGTGCGCAATATATCC | GGATATATTGCGCACGGACAG | tetracycline antibiotic |
| tet(44) | CTCATGTAGATGCAGGAAAGACG | GTAACTGCTGCCTGAATTGTGA | tetracycline antibiotic |
| tet32 | CCATTACTTCGGACAACGGTAGA | CAATCTCTGTGAGGGCATTTAACA | tetracycline antibiotic |
| tet36 | AGAATACTCAGCAGAGGTCAGTTCCT | TGGTAGGTCGATAACCCGAAAAT | tetracycline antibiotic |
| tetA | CTCACCAGCCTGACCTCGAT | CACGTTGTTATAGAAGCCGCATAG | tetracycline antibiotic |
| tetA(P) | GGAAACCTTAGTTCAGTGACTTGG | CCCATTTAACCACGCACTGAA | tetracycline antibiotic |
| tetB | AGTGCGCTTTGGATGCTGTA | AGCCCCAGTAGCTCCTGTGA | tetracycline antibiotic |
| tetB(P) | TGGGCGACAGTAGGCTTAGAA | TGACCCTACTGAAACATTAGAAATATACCT | tetracycline antibiotic |
| tetC | ACTGGTAAGGTAAACGCCATTGTC | ATGCATAAACCAGCCATTGAGTAAG | tetracycline antibiotic |
| tetD | AATTGCACTGCCTGCATTGC{EndPos:952} | GACAGATTGCCAGCAGCAGA{EndPos:1127} | tetracycline antibiotic |
| tetE | TTGGCGCTGTATGCAATGAT | CGACGACCTATGCGATCTGA | tetracycline antibiotic |
| tetG | TCGCGTTCCTGCTTGCC | CCGCGAGCGACAAACCA | tetracycline antibiotic |
| tetH | TTTGGGTCATCTTACCAGCATTAA | TTGCGCATTATCATCGACAGA | tetracycline antibiotic |
| tetJ | CAGCGCCCATACGCCATTTA | CCTACTTCAGTAGTGTGCCAAGC | tetracycline antibiotic |
| tetK | CAGCAGTCATTGGAAAATTATCTGATTATA | CCTTGTACTAACCTACCAAAAATCAAAATA | tetracycline antibiotic |
| tetL | ATGGTTGTAGTTGCGCGCTATAT | ATCGCTGGACCGACTCCTT | tetracycline antibiotic |
| tetM | GGAGCGATTACAGAATTAGGAAGC | TCCATATGTCCTGGCGTGTC | tetracycline antibiotic |
| tetO | CAACATTAACGGAAAGTTTATTGTATACCA | TTGACGCTCCAAATTCATTGTATC | tetracycline antibiotic |
| tetPB | TGGCAAGACGAGTTTGACTGA | GATCGCTCCACTTCAGCGATAA | tetracycline antibiotic |
| tetQ | CGCCTCAGAAGTAAGTTCATACACTAAG | TCGTTCATGCGGATATTATCAGAAT | tetracycline antibiotic |
| tetR | CCGTCAATGCGCTGATGAC | GCCAATCCATCGACAATCACC | tetracycline antibiotic |
| tetS | TTAAGGACAAACTTTCTGACGACATC | TGTCTCCCATTGTTCTGGTTCA | tetracycline antibiotic |
| tetT | CCATATAGAGGTTCCACCAAATCC | TGACCCTATTGGTAGTGGTTCTATTG | tetracycline antibiotic |
| tetU | GTGGCAAAGCAACGGATTG | TGCGGGCTTGCAAAACTATC | tetracycline antibiotic |
| tetW | ATGAACATTCCCACCGTTATCTTT | ATATCGGCGGAGAGCTTATCC | tetracycline antibiotic |
| tetX | AAATTTGTTACCGACACGGAAGTT | CATAGCTGAAAAAATCCAGGACAGTT | tetracycline antibiotic |
| TLA beta-lactamase | ACACTTTGCCATTGCTGTTTATGT | TGCAAATTTCGGCAATAATCTTT | beta-lactamase |
| Tn3 | GCTGAGGTGTTCAGCTACATCC | GCTGAGGTAGTCACAGGCATTC | MGE |
| TN5 | CAGCATAAAAAATCCCGACAACA | CCCCGCAACAGACATACGT | Insertional |
| TN5403 | AAGCGAATGGCGCGAAC | CGCGCAGGGTAAACTGC | MGE |
| tnpA | GCCGCACTGTCGATTTTTATC | GCGGGATCTGCCACTTCTT | Transposase |
| tnpA | CCGATCACGGAAAGCTCAAG | GGCTCGCATGACTTCGAATC | Transposase |
| tnpA | GGGCGGGTCGATTGAAA | GTGGGCGGGATCTGCTT | Transposase |
| tnpA | CATCATCGGACGGACAGAATT | GTCGGAGATGTGGGTGTAGAAAGT | Transposase |
| tnpA | GAAACCGATGCTACAATATCCAATTT | CAGCACCGTTTGCAGTGTAAG | Transposase |
| tnpA | TGCAGATGGTTTAACCTTGGATATTT | TCGGTTCATCAAACTGCTTCAC | Transposase |
| tnpA | AATTGATGCGGACGGCTTAA | TCACCAAACTGTTTATGGAGTCGTT | Transposase |
| tolC | GGCCGAGAACCTGATGCA | AGACTTACGCAATTCCGGGTTA | multidrug |
| Tp614 | GGAAATCAACGGCATCCAGTT | CATCCATGCGCTTTTGTCTCT | Transposase |
| tra-A | AAGTGTTCAGGGTGCTTCTGCGC | GTCATGTACATGATGACCAAAA | plasmid |
| traN | GCTTGGCGGTCAGCAATT | TTAGGAATAACAATCGCTACACCTTTA | plasmid |
| trb-C | CGGYATWCCGSCSACRCTGCG | GCCACCTGYSBGCAGTCMCC | plasmid |
| trfa | ACGAAGAAATGGTTGTCCTGTTC | CGTCAGCTTGCGGTACTTCTC | Transposase |
| ttgA | ACGCCAATGCCAAACGATT | GTCACGGCGCAGCTTGA | multidrug |
| ttgB | TCGCCCTGGATGTACACCTT | ACCATTGCCGACATCAACAAC | multidrug |
| vanA | GGGCTGTGAGGTCGGTTG | TTCAGTACAATGCGGCCGTTA | glycopeptide antibiotic |
| VanB | TTGTCGGCGAAGTGGATCA | AGCCTTTTTCCGGCTCGTT | glycopeptide antibiotic |
| vanC | CCTGCCACAATCGATCGTT | CGGCTTCATTCGGCTTGATA | glycopeptide antibiotic |
| vanC2_vanC3 | TGACTGTCGGTGCTTGTGA | GATAGAGCAGCTGAGCTTGTTC | glycopeptide antibiotic |
| vanD | CAGAGGAACATAATGTTTCGATAAAATCT | GCCGGATTTTGTGATTCCAA | glycopeptide antibiotic |
| vanG | TGTTTCGCAGAACCGTGTCAA | CCCTGCACTGTTCCATCTTCTC | glycopeptide antibiotic |
| vanHB | GAGGTTTCCGAGGCGACAA | CTCTCGGCGGCAGTCGTAT | glycopeptide antibiotic |
| vanHD | GTGGCCGATTATACCGTCATG | CGCAGGTCATTCAGGCAAT | glycopeptide antibiotic |
| vanRA | CCCTTACTCCCACCGAGTTTT | TTCGTCGCCCCATATCTCAT | glycopeptide antibiotic |
| vanRB | GCCCTGTCGGATGACGAA | TTACATAGTCGTCTGCCTCTGCAT | glycopeptide antibiotic |
| vanRC | TGCGGGAAAAACTGAACGA | CCCCCCATACGGTTTTGATTA | glycopeptide antibiotic |
| vanRC4 | AGTGCTTTGGCTTATCTCGAAAA | TCCGGCAGCATCACATCTAA | glycopeptide antibiotic |
| vanRD | TTATAATGGCAAGGATGCACTAAAGT | CGTCTACATCCGGAAGCATGA | glycopeptide antibiotic |
| vanSA | CGCGTCATGCTTTCAAAATTC | TCCGCAGAAAGCTCAATTTGTT | glycopeptide antibiotic |
| vanSB | GAAGATAAAGAGGGAAGCGTACTC | CCGAATTGTCAGCCCTTGATAA | glycopeptide antibiotic |
| vanSC | ATCAACTGCGGGAGAAAAGTCT | TCCGCTGTTCCGCTTCTT | glycopeptide antibiotic |
| vanTC | ACAGTTGCCGCTGGTGAAG | CGTGGCTGGTCGATCAAAA | glycopeptide antibiotic |
| vanTE | GTGGTGCCAAGGAAGTTGCT | CGTAGCCACCGCAAAAAAAT | glycopeptide antibiotic |
| vanTG | CGTGTAGCCGTTCCGTTCTT | CGGCATTACAGGTATATCTGGAAA | glycopeptide antibiotic |
| vanWB | CGGACAAAGATACCCCCTATAAAG | AAATAGTAAATTGCTCATCTGGCACAT | glycopeptide antibiotic |
| vanXA | TCGTTGGGACGCTAAATATGC | GGACGGTAACCGTCCCATA | glycopeptide antibiotic |
| vanXB | AGGCACAAAATCGAAGATGCTT | GGGTATGGCTCATCAATCAACTT | glycopeptide antibiotic |
| vanYB | GGCTAAAGCGGAAGCAGAAA | GATATCCACAGCAAGACCAAGCT | glycopeptide antibiotic |
| vanYD | AAGGCGATACCCTGACTGTCA | ATTGCCGGACGGAAGCA | glycopeptide antibiotic |
| vatA | ATGAACGGAGCGAATCATCGG | CCATACCGATCCAAACGTCATTTC | MLSB |
| vatB | GCAATTGTTGCTGCGAATTCAG | GTGCTGACCAATCCCACCA | MLSB |
| vatE | GACCGTCCTACCAGGCGTAA | TTGGATTGCCACCGACAATT | MLSB |
| VEB beta-lactamase | CCCGATGCAAAGCGTTATG | GAAAGATTCCCTTTATCTATCTCAGACAA | beta-lactamase |
| vgaA | GGAAGCTATAGAGGCGTTTGAATC | CCGAAGGTTCAATACTCAATCGAC | Multidrug |
| vgaALC | GTGAAGATGTCTCGGGTACAATTG | GAAATACCAGGATTCCCATGCAC | Multidrug |
| vgaB | TAAAAGAGAATAAGGCGCAAGGA | TGTTTAGTAGCATGTTGCATTTTCC | Multidrug |
| VIM beta-lactamase | GCACTTCTCGCGGAGATTG | CGACGGTGATGCGTACGTT | beta-lactamase |

**Table S3** The number of ARGs and VFGs located on *Rhizobiales*

| Genome | Taxon | Geographic location | Environment | ARGs | VFGs |
| --- | --- | --- | --- | --- | --- |
| GCA_000421645.1 | *Aurantimonas coralicida DSM 14790* | USA | Marine | 1 | 15 |
| GCA_000960975.1 | *Martelella endophytica YC6887* | Korea | Plant | 0 | 6 |
| GCA_001463945.1 | *Aureimonas ureilytica DSM 18598 NBRC 106430* | South Korea | Air | 0 | 3 |
| GCA_002043005.1 | *Martelella mediterranea DSM 17316* | Spain | Water | 0 | 6 |
| GCA_005924265.1 | *Martelella lutilitoris GH2-6* | South Korea | Rhizosphere | 0 | 12 |
| GCA_014058705.1 | *Aureimonas mangrovi LMG 31693* | Thailand | Mangrove sediment | 0 | 3 |
| GCA_017815515.1 | *Aureimonas populi KCTC 42087* | China | Plant | 0 | 3 |
| GCA_024105745.1 | *Aurantimonas endophytica KCTC 52296* | China | NA | 0 | 3 |
| GCA_026898155.1 | *Jiella pelagia HL-NP1* | Pacific Ocean | Water | 0 | 3 |
| GCA_900141975.1 | *Aureimonas altamirensis DSM 21988* | Spain | Cave | 0 | 3 |
| GCA_000196435.1 | *Bartonella tribocorum CIP 105476* | France | Animal | 0 | 140 |
| GCA_000341355.1 | *Bartonella australis AUST/NH1* | Australia | Animal | 0 | 12 |
| GCA_001281405.1 | *Bartonella ancashensis 20* | Peru | Human | 0 | 30 |
| GCA_003606325.3 | *Bartonella kosoyi Tel Aviv* | Israel | Animal | 0 | 105 |
| GCA_003606345.3 | *Bartonella krasnovii OE 1-1* | Israel | Animal | 0 | 77 |
| GCA_009498695.1 | *Bartonella bacilliformis KC584* | France | Human | 0 | 36 |
| GCA_009936175.1 | *Bartonella quintana MF1-1* | Japan | Animal | 0 | 108 |
| GCA_013388295.1 | *Bartonella alsatica CIP 105477* | France | Animal | 0 | 62 |
| GCA_024297065.1 | *Bartonella harrusi 117A* | Brazil | Animal | 0 | 60 |
| GCA_000019845.1 | *Beijerinckia indica subsp. indica ATCC 9039* | NA | NA | 0 | 1 |
| GCA_000021745.1 | *Methylocella silvestris BL2* | NA | NA | 0 | 2 |
| GCA_000385335.1 | *Methyloferula stellata AR4* | USA | Water | 0 | 2 |
| GCA_000427445.1 | *Methylocapsa acidiphila B2* | NA | Bog | 0 | 2 |
| GCA_000745425.1 | *Beijerinckia mobilis UQM 1969* | NA | NA | 0 | 18 |
| GCA_000746085.1 | *Methylocapsa aurea KYG* | NA | NA | 0 | 2 |
| GCA_004135935.1 | *Methylovirgula ligni BW863* | NA | Soil | 0 | 2 |
| GCA_900114285.1 | *Methylocapsa palsarum NE2* | NA | NA | 0 | 2 |
| GCA_901905185.2 | *Methylocella tundrae 2* | USA | Water and Sediments | 0 | 2 |
| GCA_000012725.1 | *Nitrobacter winogradskyi Nb-255* | NA | NA | 0 | 3 |
| GCA_000013885.1 | *Nitrobacter hamburgensis X14* | NA | NA | 0 | 3 |
| GCA_000218565.1 | *Afipia carboxidovorans OM5* | Germany | Wastewater | 0 | 3 |
| GCA_000344805.1 | *Bradyrhizobium oligotrophicum S58* | NA | Soil | 0 | 3 |
| GCA_002266435.3 | *Bradyrhizobium amphicarpaeae 39S1MB* | Canada | Plantt | 0 | 3 |
| GCA_002355335.1 | *Variibacter gotjawalensis GJW-30* | Korea | Soil | 0 | 2 |
| GCA_004114975.1 | *Bradyrhizobium guangdongense CCBAU 51649* | China | Plant | 1 | 4 |
| GCA_008932115.1 | *Bradyrhizobium betae PL7HG1* | Spain | Plant | 1 | 3 |
| GCA_015291705.1 | *Bradyrhizobium arachidis CCBAU 051107* | China | Plant | 1 | 5 |
| GCA_021044685.1 | *Bradyrhizobium daqingense CCBAU 15774* | China | Plant | 1 | 6 |
| GCA_000007125.1 | *Brucella melitensis bv. 1 str. 16M* | NA | Animal | 3 | 269 |
| GCA_000007505.1 | *Brucella suis 1330* | NA | Animal | 3 | 264 |
| GCA_000016845.1 | *Brucella ovis ATCC 25840* | NA | Animal | 3 | 278 |
| GCA_000022745.1 | *Brucella microti CCM 4915* | NA | Animal | 3 | 259 |
| GCA_000054005.1 | *Brucella abortus 2308* | USA | Animal | 3 | 266 |
| GCA_000590795.1 | *Brucella ceti TE10759-12* | Mediterranean Sea | Animal | 3 | 261 |
| GCA_002278035.1 | *[Ochrobactrum] quorumnocens A44* | Netherlands | Rhizosphere | 3 | 62 |
| GCA_018436245.1 | *Pseudochrobactrum algeriensis C130915_07* | Algeria | Animal | 1 | 17 |
| GCA_002866925.1 | *Cohaesibacter celericrescens H1304* | China | Animal | 0 | 2 |
| GCA_003324485.1 | *Cohaesibacter intestini YE-B6* | China | Animal | 0 | 3 |
| GCA_003574655.1 | *Cohaesibacter haloalkalitolerans JC131* | India | Water | 0 | 2 |
| GCA_900115225.1 | *Cohaesibacter marisflavi CGMCC 1.9157* | NA | NA | 0 | 17 |
| GCA_900215605.1 | *Cohaesibacter gelatinilyticus DSM 18289* | NA | NA | 0 | 2 |
| GCA_000143145.1 | *Hyphomicrobium denitrificans ATCC 51888* | NA | Water | 0 | 1 |
| GCA_000166055.1 | *Rhodomicrobium vannielii ATCC 17100* | NA | NA | 0 | 1 |
| GCA_000383415.1 | *Hyphomicrobium zavarzinii ATCC 27496* | Russia | Soil | 0 | 1 |
| GCA_000503895.1 | *Hyphomicrobium nitrativorans NL23* | Canada | Water | 0 | 1 |
| GCA_000828475.1 | *Methyloceanibacter caenitepidi Gela4* | Japan | Sediment | 0 | 1 |
| GCA_003258865.1 | *Rhodoplanes roseus DSM 5909* | NA | NA | 0 | 3 |
| GCA_003550175.1 | *Dichotomicrobium thermohalophilum DSM 5002* | NA | NA | 0 | 1 |
| GCA_009708035.1 | *Hyphomicrobium album XQ2* | China | Soil | 0 | 1 |
| GCA_013306565.1 | *Hyphomicrobium sulfonivorans S1* | United Kingdom | Soil | 0 | 1 |
| GCA_030813915.1 | *Rhodoplanes tepidamans DSM 9987* | NA | NA | 0 | 2 |
| GCA_000019725.1 | *Methylobacterium radiotolerans JCM 2831* | NA | Plant | 1 | 3 |
| GCA_000019945.1 | *Methylorubrum populi BJ001* | USA | Plant | 4 | 3 |
| GCA_000022085.1 | *Methylobacterium nodulans ORS 2060* | Senegal | Plant | 0 | 3 |
| GCA_000083545.1 | *Methylorubrum extorquens DM4* | NA | NA | 4 | 3 |
| GCA_000757795.1 | *Methylobacterium oryzae CBMB20* | Korea | Phyllosphere | 1 | 3 |
| GCA_001936175.1 | *Methylobacterium phyllosphaerae CBMB27* | South Korea | Phyllosphere | 1 | 3 |
| GCA_002741015.1 | *Microvirga ossetica V5/3m* | Russia | Plant nodules | 0 | 2 |
| GCA_003173715.1 | *Methylobacterium durans 17SD2-17* | South Korea | Soil | 2 | 3 |
| GCA_003173755.1 | *Methylobacterium terrae 17Sr1-28* | South Korea | Soil | 0 | 2 |
| GCA_000178815.2 | *Methylosinus trichosporium OB3b* | Canada | Soil | 0 | 2 |
| GCA_000372845.1 | *Methylocystis rosea SV97* | USA | MIGS Cultured Bacterial | 0 | 2 |
| GCA_002117405.1 | *Methylocystis bryophila S285* | Russia | Soil | 0 | 2 |
| GCA_003113265.1 | *Methylosinus sporium DSM 17706* | NA | Water | 0 | 1 |
| GCA_003722355.1 | *Methylocystis hirsuta CSC1* | USA | Water | 0 | 2 |
| GCA_004802635.2 | *Methylocystis heyeri H2* | Germany | Water | 0 | 2 |
| GCA_009685195.1 | *Methylocystis parvus BRCS2* | United Kingdom | Bog sediment | 0 | 2 |
| GCA_013350005.1 | *Methylocystis silviterrae FS* | Russia | Soil | 0 | 8 |
| GCA_024448135.1 | *Methylocystis suflitae NLS-7* | USA | Soil | 0 | 2 |
| GCA_027925385.1 | *Methylocystis iwaonis SS37A-Re* | Japan | Soil | 0 | 2 |
| GCA_000176035.2 | *Mesorhizobium opportunistum WSM2075* | Australia | Rhizosphere | 0 | 7 |
| GCA_000230995.3 | *Mesorhizobium australicum WSM2073* | Australia | Plant | 0 | 7 |
| GCA_001889605.1 | *Aquibium oceanicum B7* | China | Water | 0 | 3 |
| GCA_002764115.1 | *Phyllobacterium zundukense Tri-48; RCAM 03910* | Russia | Root nodule | 0 | 6 |
| GCA_007922615.2 | *Nitratireductor mangrovi SY7* | China | Soil | 0 | 3 |
| GCA_008727715.1 | *Mesorhizobium terrae NIBRBAC000500504* | South Korea | Soil | 0 | 3 |
| GCA_013170785.1 | *Mesorhizobium jarvisii ATCC 33669* | New Zealand | Plant | 0 | 11 |
| GCA_014495845.1 | *Aquibium microcysteis NIBR3* | South Korea | Water | 0 | 3 |
| GCA_024707545.1 | *Mesorhizobium onobrychidis* | Germany | Plant | 0 | 31 |
| GCA_036621415.1 | *Nitratireductor thuwali Nit1536* | Saudi Arabia | Sediments | 0 | 3 |
| GCA_000009265.1 | *Rhizobium johnstonii 3841* | NA | Plant | 0 | 14 |
| GCA_000330885.1 | *Rhizobium tropici CIAT 899* | Colombia | Root nodule | 0 | 6 |
| GCA_000697965.2 | *Ensifer adhaerens Casida A* | USA | Soil | 0 | 118 |
| GCA_000731315.1 | *Neorhizobium galegae bv. orientalis str. HAMBI 540* | Finland | Root nodule | 0 | 8 |
| GCA_013488225.1 | *Sinorhizobium mexicanum ITTG R7* | Mexico | Plant | 0 | 15 |
| GCA_017352135.1 | *Rhizobium lentis BLR27* | China | Plant | 0 | 9 |
| GCA_017357225.1 | *Rhizobium binae BLR195* | China | Plant | 0 | 3 |
| GCA_020883495.1 | *Shinella zoogloeoides ATCC 19623* | China | Activated sludge | 2 | 3 |
| GCA_034554815.1 | *Rhizobium indigoferae CIP 108029* | China | Plant | 0 | 3 |
| GCA_902502825.2 | *Pseudorhizobium flavum YW14* | China | Soil | 0 | 5 |
